# The growing burden of generalized myasthenia gravis: a population-based retrospective cohort study in Taiwan

**DOI:** 10.3389/fneur.2023.1203679

**Published:** 2023-06-23

**Authors:** Keira Joann Herr, Shih-Pei Shen, Yanfang Liu, Chih-Chao Yang, Chao-Hsiun Tang

**Affiliations:** ^1^Janssen Medical Affairs Asia Pacific, Singapore, Singapore; ^2^School of Health Care Administration, College of Management, Taipei Medical University, Taipei, Taiwan; ^3^Global Real-World Evidence, GCDS, GCSO, Janssen Research and Development LLC, Raritan, NJ, United States; ^4^Department of Neurology, National Taiwan University Hospital, Taipei, Taiwan

**Keywords:** database, epidemiology, myasthenia gravis, Taiwan, hepatitis B, incidence, prevalence

## Abstract

**Background:**

The prevalence of myasthenia gravis is increasing in many countries, including Asia. As treatment options expand, population-based information about the disease burden can inform health technology assessments.

**Methods:**

We conducted a population-based retrospective cohort study using the Taiwan National Healthcare Insurance Research database and Death Registry to describe the epidemiology, disease burden and treatment patterns of generalized myasthenia gravis (gMG) from 2009 to 2019. Episodes of hepatitis B virus (HBV) infection or reactivation were explored.

**Results:**

The number of patients with gMG increased from 1,576 in 2009 to 2,638 in 2019 and the mean (standard deviation) age from 51.63 (17.32) to 55.38 (16.29) years. The female:male ratio was 1.3:1. Frequently reported co-morbidities were hypertension (32–34% of patients), diabetes mellitus (16–21%) and malignancies (12–17%). The prevalence of patients with gMG increased annually from 6.83/100,000 population in 2009 to 11.18/100,000 population in 2019 (*p* < 0.0001). There was no temporal trend in all-cause fatality rates (range 2.76–3.79/100 patients annually) or gMG incidence rates (2.4–3.17/100,000 population annually). First-line treatment was with pyridostigmine (82%), steroids (58%), and azathioprine (11%). There was minimal change in treatment patterns over time. Among 147 new HBV infections, 32 (22%) received ≥4 weeks of antiviral therapy suggesting chronic infection. The HBV reactivation rate was 7.2%.

**Conclusion:**

The epidemiology of gMG in Taiwan is evolving rapidly, with higher prevalence rates and increasing involvement of older age-groups suggesting a growing burden of disease and associated healthcare costs. HBV infection or reactivation may pose a previously unrecognized recognized risk for patients with gMG receiving immunosuppressants.

## 1. Introduction

Myasthenia gravis (MG) is the most common disorder of the neuromuscular junction and encompasses a group of diseases with a wide range of symptoms that usually include muscle weakness and fatigue. MG may be congenital or acquired and is mediated by B cells that produce antibodies directed at post-synaptic components of the endplate of voluntary muscles. Patients with antibodies against acetylcholine receptors represent the largest group, but other self-antigens include muscle-specific kinase (MuSK) and lipoprotein-associated protein (1). Ocular weakness is the most common presenting symptom, and around 50% of patients develop generalized MG (gMG) within 2 years of diagnosis (2, 3). The heterogenous disease etiology and clinical presentation, coupled with observed regional and ethnic differences in epidemiology, suggest that MG may represent a common presentation of many different diseases (1, 2).

Initial treatment for gMG is usually with anticholinesterase inhibitors and thymectomy when indicated, with immunosuppression using corticosteroids if symptoms persist (4). Corticosteroids are highly effective in gMG but due to their potential for serious side-effects, are frequently administered with immunosuppressive drugs, such as azathioprine, mycophenolate mofetil, cyclosporine, cyclophosphamide, or methotrexate as steroid-sparing agents (4). Intravenous immunoglobulin is also a treatment option for some patients (5). Patients with refractory and treatment-resistant gMG may be considered for plasma exchange and monoclonal antibodies such as rituximab; a monoclonal antibody directed against CD20 B cells; and eculizumab, a monoclonal antibody against terminal C5 complement currently recommended for use in patients with severe refractory gMG (5).

The rarity of MG hinders a clear understanding of its epidemiology in different populations. A systematic review of the literature until 2008 reported MG incidence rates ranging from 1.7 to 21.3 per million person-years, with large variations in age and sex distribution between studies (6). In some studies the incidence of MG in women was characterized by a bimodal peak at 29-39-years and after 60 years of age (6). All studies showed an increase in the incidence of MG in men and women with age (6). The incidence of MG has increased over time, attributed to improved testing, better study quality, aging populations and their associated immunological milieu, and/or environmental factors (6, 7).

The epidemiology of MG in Asian countries/regions is not well understood. Incidence rates of 4 and 4.5 per million person-years have been reported in Hong Kong and Japan respectively, with higher proportions of congenital MG than in other regions (6). A nationwide study of patients with MG who received hospital care in China reported an incidence of 6.8 per million person-years, with a higher incidence in females than males (7.6 vs. 6.0 per million person-years) (8). A more recent study from Japan confirmed a high rate of infantile-onset disease and noted marked heterogeneity in clinical presentation according to age of onset (9).

Treatment options for gMG are expanding as experience with monoclonal antibodies grows, and new drugs, such as neonatal Fc receptor antibodies (nipocalimab and efgartigimod) are in development or emerging in the market. New treatments for gMG will need health technology assessments supported by reference population-based information about the disease burden. Relatively high rates of hepatitis B (HBV) infection in some Asian countries could impact the use of new treatments that result in chronic immunosuppression and an increased risk of virus reactivation (10). In Taiwan, the seropositivity rate for HBV surface antigen (HBsAg) in the general population was 13.7% in 2002 (11). It is important to know the prevalence of HBV infection/reactivation when treating gMG, particularly in Asian countries where the prevalence of chronic hepatitis B in adults continues to be high.

We conducted a retrospective population-based cohort study using the National Health Insurance Research Database (NHIRD) in Taiwan. The study objectives were to estimate incidence, prevalence, all-cause mortality and treatment patterns in patients with gMG in the Taiwanese population. In view of the high burden of HBV infection in Taiwanese adults (11), we also assessed the prevalence of HBV reactivation after diagnosis with gMG given that many of these patients may be receiving immunosuppressing drugs.

The data from this real-world study will help inform the burden of disease, identify unmet medical needs, and help shape the disease management algorithm for patients with gMG. Understanding patterns of use of currently available treatments will facilitate optimal care for patients and contribute to the introduction of new treatments that could improve the clinical outcomes of patients with gMG.

## 2. Methods

### 2.1. Data source

The NHIRD is a population-based, longitudinal electronic claims database in Taiwan established in 1995 that captures all health-related claims data for approximately 99% of the population (12). The database holds all information including diagnoses, treatments, inpatient, outpatient, and pharmaceutical services administered across the country. Diagnoses were coded using International Classification of Diseases (ICD), Ninth Revision, Clinical Modification (ICD-9-CM) until 2016, and the tenth version (ICD-10) thereafter. NHIRD-specific drug codes capture the drug, administration route and dose prescribed (but not the dose actually administered). The NHIRD database is maintained by the Data Science Center which is a part of the Ministry of Health and Welfare in Taiwan. Personal information is anonymized and personal identifiers including name and date of birth are de-identified in the database to maintain data privacy.

The Taiwan National Death Registry is managed separately to the NHIRD and data are linked at the patient level using scrambled identification codes. It is mandatory to report all deaths to the Death Registry within 28 days of the event using ICD codes. All death certificates issued by doctors in Taiwan are reviewed centrally by trained medical personnel, and coding of cause of death is considered highly accurate and complete in Taiwan (13).

This study was granted an exemption from the need for patient consent and from ethical review by the Taipei Medical University-Joint Institutional Review Board. The study was conducted according to all applicable guidelines and regulations set by the Health and Welfare Data Science Center. All analyses used de-identified, aggregated patient data.

### 2.2. Study population

The study cohort comprised all patients in the NHIRD with a diagnosis of MG (ocular or general) (ICD-9358.00, 358.0; ICD-10 G70.00, G70.01) between January 01, 2009 and December 31, 2019. All individuals with MG were identified in the NHIRD using ICD codes plus evidence of at least three outpatient visits or one hospital admission claim with an MG diagnosis code within 1 year, with treatment of MG at all of the three outpatient or inpatient visits.

Patients were considered to have gMG if they were an inpatient for MG as the main or sub-diagnosis under any discipline, excluding patients with ophthalmologic diagnoses (main or sub-diagnosis) alone; or if they had received treatment with azathioprine during the study; or if they had received at least one treatment period of steroids prescribed daily at a minimum dose of 20 mg prednisolone (or equivalent) for 28 days. These criteria reflect current practice in Taiwan where patients with ocular MG are treated with low-dose steroids and infrequently receive azathioprine, and ensured that we captured the majority of gMG cases.

The diagnosis index date was defined as the first date of diagnosis of gMG in the NHIRD between 2009 and 2019. Incident cases were patients with a first diagnosis of gMG between 2010 to 2019 who had no preceding gMG diagnosis from January 01, 2009. The baseline period was defined as 12 months prior to the diagnosis index date and was used to assess the presence of co-morbidities. All patients were followed from the diagnosis index date until the date of study end (December 31, 2019) or until death or dis-enrollment, whichever occurred first.

### 2.3. Outcomes

Demographic features of patients, index year of diagnosis, and the presence of co-morbidities during the baseline period were assessed. Co-morbidities were identified using ICD9/ICD10 codes and required at least two outpatient visits and/or one inpatient hospitalization record, and/or one emergency room visit to be confirmed.

Patients with HBV and hepatitis C virus (HCV) infection were identified using ICD codes and evidence of an antiviral treatment prescription. Active HCV infection was defined as diagnosis of HCV infection in a patient who received antiviral treatment for at least 4 weeks. HBV infection was defined as a diagnosis of HBV infection (at least two consecutive outpatient visits and/or one inpatient visit) during the study period. An active HBV infection was defined as a diagnosis of HBV infection and receiving antiviral treatment for at least 4 weeks. HBV reactivation was defined as patients who received antiviral treatment for a diagnosis of HBV infection after discontinuation of antiviral treatment for at least 6 months.

Treatments for gMG, HBV, and HCV were identified using Anatomical Therapeutic Chemical codes. The treatment regimens, treatment patterns, lines of treatment and duration of treatment were recorded. The first treatment administered was considered to be first line treatment. If a new treatment (different class of drug) was added, this was considered a new line of treatment. If a treatment was discontinued for at least 3 months it was considered that the line of treatment had ceased.

### 2.4. Statistical analysis

The annual incidence rate of gMG by calendar year was estimated by dividing the total number of patients with newly diagnosed gMG in each year by the total number of persons at mid-year. The annual prevalence rate of gMG by calendar year was calculated by dividing the total number of patients diagnosed with gMG in each year by the total number of persons at mid-year. Trends in prevalence rates were assessed using the two-sided Cochran-Armitage test. Annual all-cause mortality was calculated by dividing the total number of deaths in each year by the total number of patients with gMG in each calendar year. Age and gender-specific incidence and prevalence rates were calculated with exact 95% confidence intervals (CI). The number and percentages of patients with HBV and HCV infection and the rate of HBV reactivation were calculated with exact 95% CIs.

## 3. Results

The total number of patients with MG (ocular or generalized) increased from 3,647 in 2009 to 6,293 in 2019, with prevalence rates that increased annually from 15.80 per 100,000 population to 26.67 per 100,000 population, a 1.7-fold increase over the study period (Supplementary Table S1, *p* < 0.001 2-sided Cochran-Armitage test). There was evidence that over time, more patients with gMG survived into older age. For example, the number of individuals aged ≥80 years in Taiwan increased from 552,645 in 2009 to 803,856 in 2019, and the number of patients with gMG in this age group increased from 76 to 175 (Supplementary Table S2).

The number of patients with gMG increased from 1,576 in 2009 to 2,638 in 2019 but the percentage of all GM patients with gMG remained steady at 40–43% annually (Table 1). The mean age of patients increased annually in each study year, from 51.63 [standard deviation (SD) 17.32] in 2009 to 55.38 (SD 16.29) in 2019. No more than 10% of patients with gMG were < 30 years of age in any study year. Females exceeded males by approximately 1.3:1. The most frequently reported co-morbidities were hypertension (present in 31.6 to 33.78% of patients), type 1 or 2 diabetes mellitus (16.62–21.77%) and malignancies (12.18–16.66%).

**Table 1:** Demographic features and co-morbidities in patients diagnosed with generalized myasthenia gravis in Taiwan from 2009 to 2019 (Prevalent cohort).

Category	Year
	2009 *n* = 1,576	2010 *n* = 1,697	2011 *n* = 1793	2012 *n* = 1774	2013 *n* = 1924	2014 *n* = 2,101	2015 *n* = 2,152	2016 *n* = 2,247	2017 *n* = 2,350	2018 *n* = 2,521	2019 *n* = 2,638
Sex *n* (%)											
Male	682 (43.27)	723 (42.6)	776 (43.28)	771 (43.46)	824 (42.83)	923 (43.93)	921 (42.8)	949 (42.23)	1,023 (43.53)	1,109 (43.99)	1,158 (43.9)
Female	887 (56.28)	966 (56.92)	1,012 (56.44)	994 (56.03)	1,091 (56.7)	1,167 (55.54)	1,225 (56.92)	1,289 (57.37)	1,322 (56.26)	1,400 (55.53)	1,470 (55.72)
Age (years) *n* (%)											
≤ 9	20 (1.27)	19 (1.12)	14 (0.78)	13 (0.73)	10 (0.52)	15 (0.71)	15 (0.7)	14 (0.62)	10 (0.43)	12 (0.48)	9 (0.34)
10–19	28 (1.78)	23 (1.36)	28 (1.56)	24 (1.35)	31 (1.61)	34 (1.62)	30 (1.39)	35 (1.56)	35 (1.49)	48 (1.9)	42 (1.59)
20–29	117 (7.42)	118 (6.95)	107 (5.97)	100 (5.64)	89 (4.63)	117 (5.57)	93 (4.32)	91 (4.05)	109 (4.64)	116 (4.6)	115 (4.36)
30–39	220 (13.96)	237 (13.97)	262 (14.61)	262 (14.77)	291 (15.12)	316 (15.04)	305 (14.17)	314 (13.97)	281 (11.96)	293 (11.62)	287 (10.88)
40–49	339 (21.51)	336 (19.8)	335 (18.68)	328 (18.49)	354 (18.4)	357 (16.99)	373 (17.33)	378 (16.82)	405 (17.23)	428 (16.98)	461 (17.48)
50–59	328 (20.81)	372 (21.92)	402 (22.42)	411 (23.17)	425 (22.09)	468 (22.28)	488 (22.68)	527 (23.45)	532 (22.64)	540 (21.42)	584 (22.14)
60–69	247 (15.67)	274 (16.15)	287 (16.01)	275 (15.5)	328 (17.05)	396 (18.85)	450 (20.91)	471 (20.96)	526 (22.38)	587 (23.28)	619 (23.46)
70–79	201 (12.75)	225 (13.26)	250 (13.94)	240 (13.53)	273 (14.19)	261 (12.42)	259 (12.04)	273 (12.15)	293 (12.47)	323 (12.81)	344 (13.04)
≥ 80	76 (4.82)	93 (5.48)	108 (6.02)	121 (6.82)	123 (6.39)	137 (6.52)	139 (6.46)	144 (6.41)	159 (6.77)	174 (6.9)	175 (6.63)
Mean (±SD)	51.63 (17.32)	52.45 (17.38)	53.01 (17.25)	53.27 (17.22)	53.73 (17.03)	53.58 (17.24)	54.33 (16.85)	54.48 (16.54)	55.12 (16.4)	55.16 (16.65)	55.38 (16.29)
Co-morbidities *n* (%)											
Hypertension	498 (31.60)	555 (32.7)	570 (31.79)	579 (32.64)	643 (33.42)	720 (34.27)	726 (33.74)	759 (33.78)	782 (33.28)	834 (33.08)	881 (33.4)
Diabetes mellitus: type 1 or 2	262 (16.62)	332 (19.56)	331 (18.46)	317 (17.87)	366 (19.02)	418 (19.9)	447 (20.77)	413 (18.38)	438 (18.64)	488 (19.36)	498 (18.88)
Malignancies	192 (12.18)	225 (13.26)	247 (13.78)	239 (13.47)	288 (14.97)	318 (15.14)	338 (15.71)	371 (16.51)	383 (16.3)	420 (16.66)	439 (16.64)
Cardiovascular disease	173 (10.98)	210 (12.37)	230 (12.83)	228 (12.85)	232 (12.06)	234 (11.14)	266 (12.36)	260 (11.57)	251 (10.68)	300 (11.9)	308 (11.68)
Thyroid related conditions	87 (5.52)	113 (6.66)	115 (6.41)	125 (7.05)	119 (6.19)	147 (7)	142 (6.6)	167 (7.43)	155 (6.6)	144 (5.71)	158 (5.99)
Respiratory illness/disorder	130 (8.25)	159 (9.37)	146 (8.14)	172 (9.7)	186 (9.67)	212 (10.09)	222 (10.32)	100 (4.45)	111 (4.72)	123 (4.88)	123 (4.66)
Neurotic disorder	69 (4.38)	77 (4.54)	79 (4.41)	83 (4.68)	95 (4.94)	100 (4.76)	132 (6.13)	99 (4.41)	114 (4.85)	130 (5.16)	123 (4.66)
Renal conditions	37 (2.35)	42 (2.47)	62 (3.46)	69 (3.89)	76 (3.95)	82 (3.9)	108 (5.02)	94 (4.18)	109 (4.64)	112 (4.44)	107 (4.06)
HCV	34 (2.16)	29 (1.71)	36 (2.01)	26 (1.47)	33 (1.72)	29 (1.38)	39 (1.81)	35 (1.56)	41 (1.74)	37 (1.47)	34 (1.29)
Obesity	4 (0.25)	4 (0.24)	0 (0)	≤3	7 (0.36)	4 (0.19)	7 (0.33)	≤3	8 (0.34)	12 (0.48)	7 (0.27)
Liver abnormalities	≤3	≤3	5 (0.28)	7 (0.39)	≤3	4 (0.19)	5 (0.23)	8 (0.36)	≤3	5 (0.2)	4 (0.15)

HCV, hepatitis C virus infection; SD, standard deviation.

### 3.1. Prevalence, incidence, and all-cause fatality rates

**Figure 1:**
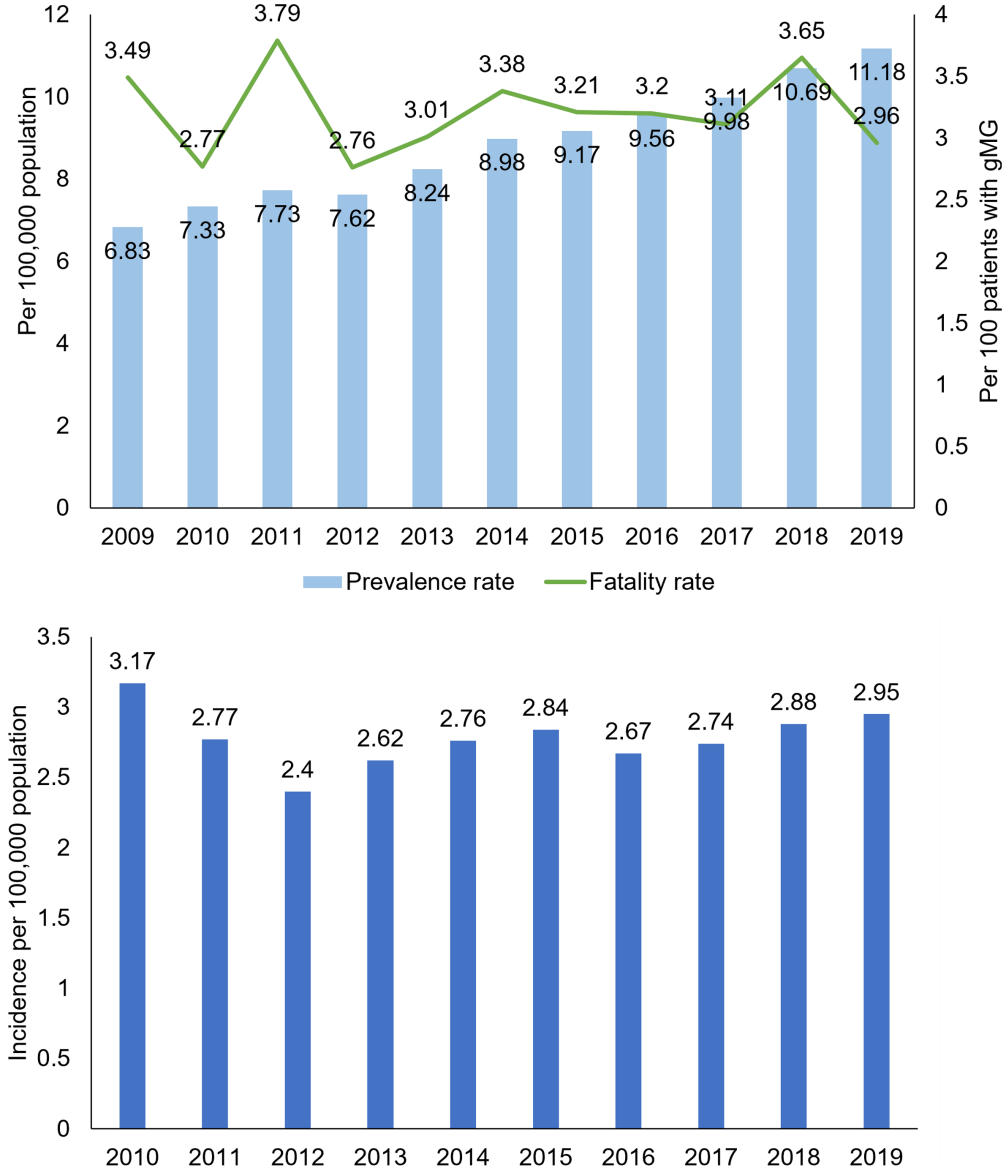
Crude prevalence, all-cause fatality, and incidence rates in patients with generalized myasthenia gravis, Taiwan 2009–2019. gMG, generalized myasthenia gravis. Data are tabulated in Supplementary Tables S2, S4.

The rate of prevalent gMG cases increased annually, from 6.83 per 100,000 population in 2019, almost doubling to 11.18 per 100,000 population in 2019 (value of *p* for trend <0.0001; Figure 1). The all-cause fatality rate ranged from 2.76 to 3.79 per 100 patients in each study year, with no consistent temporal trend observed. Similarly, incidence rates of gMG changed little over time, ranging from 2.4 and 3.17 per 100,000 population annually (Figure 1).

**Figure 2:**
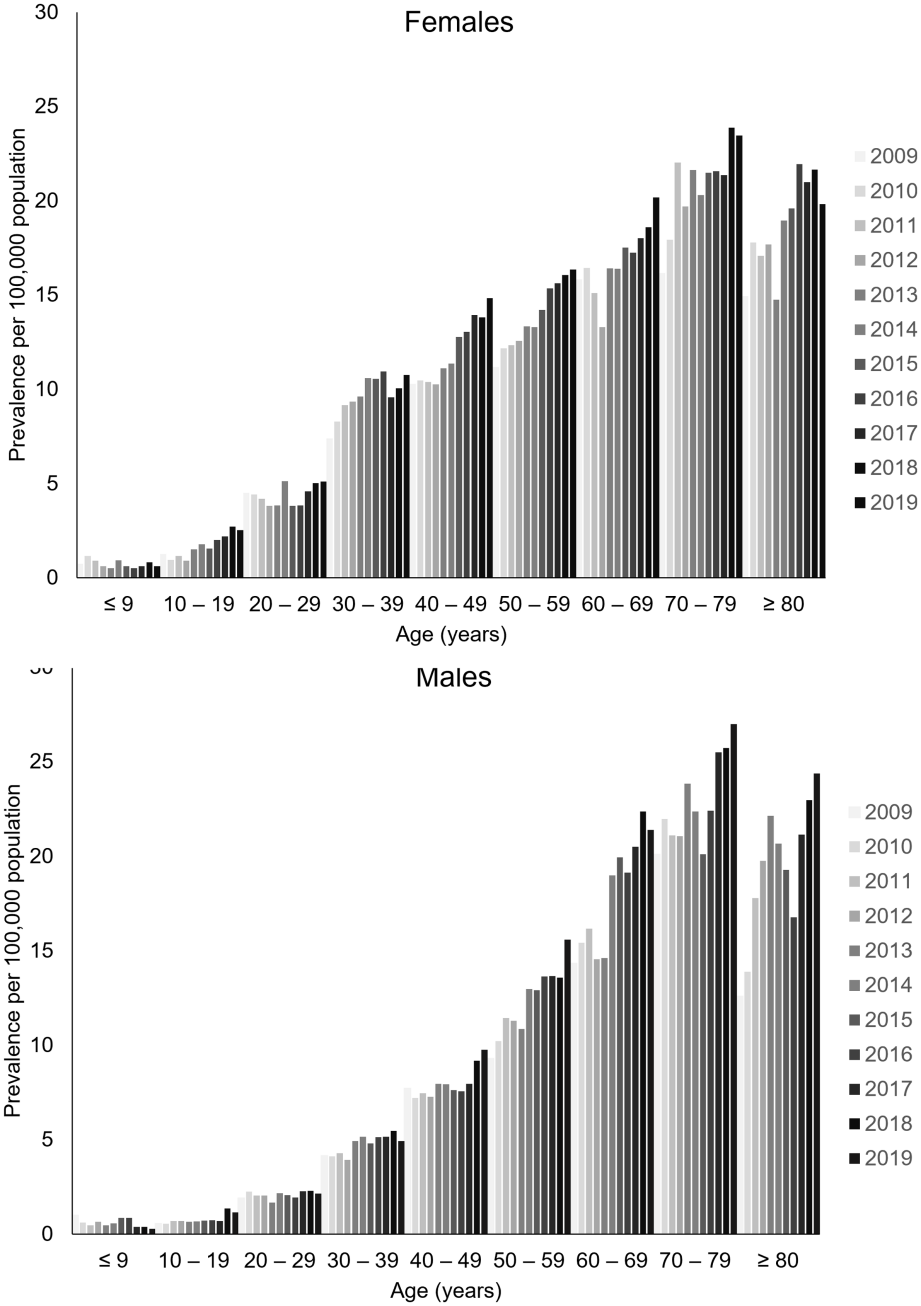
Sex-specific and age-specific prevalence of generalized myasthenia gravis in Taiwan, 2009–2019. Data are tabulated in Supplementary Table S2.

Prevalence rates, all-cause fatality rates, and incidence rates all increased markedly with age in men and women. Prevalence rates were higher in younger women than younger men, with a pronounced increase in gMG prevalence in women at 30–49 years of age that was not observed in men (Figure 2). Over time, the annual prevalence rate of gMG remained stable in women until aged 30, and in men until age 40, with increasing annual prevalence rates in most study years thereafter. In older age groups, prevalence rates tended to be similar in both sexes.

The incidence rate of gMG tended to be higher in women than in men until around 60 years of age (Supplementary Table S3). Thereafter, incidence rates were generally higher in men. There was no consistent trend in annual incidence rates suggesting any change in the incidence rate of gMG over time in any age-group, or in men or women.

All-cause fatality rates varied considerably in men and women from year to year (Supplementary Table S4). Overall, all cause fatality rates appeared to be similar in men and women, increasing markedly from 80 years of age.

### 3.2. Treatment patterns

**Figure 3:**
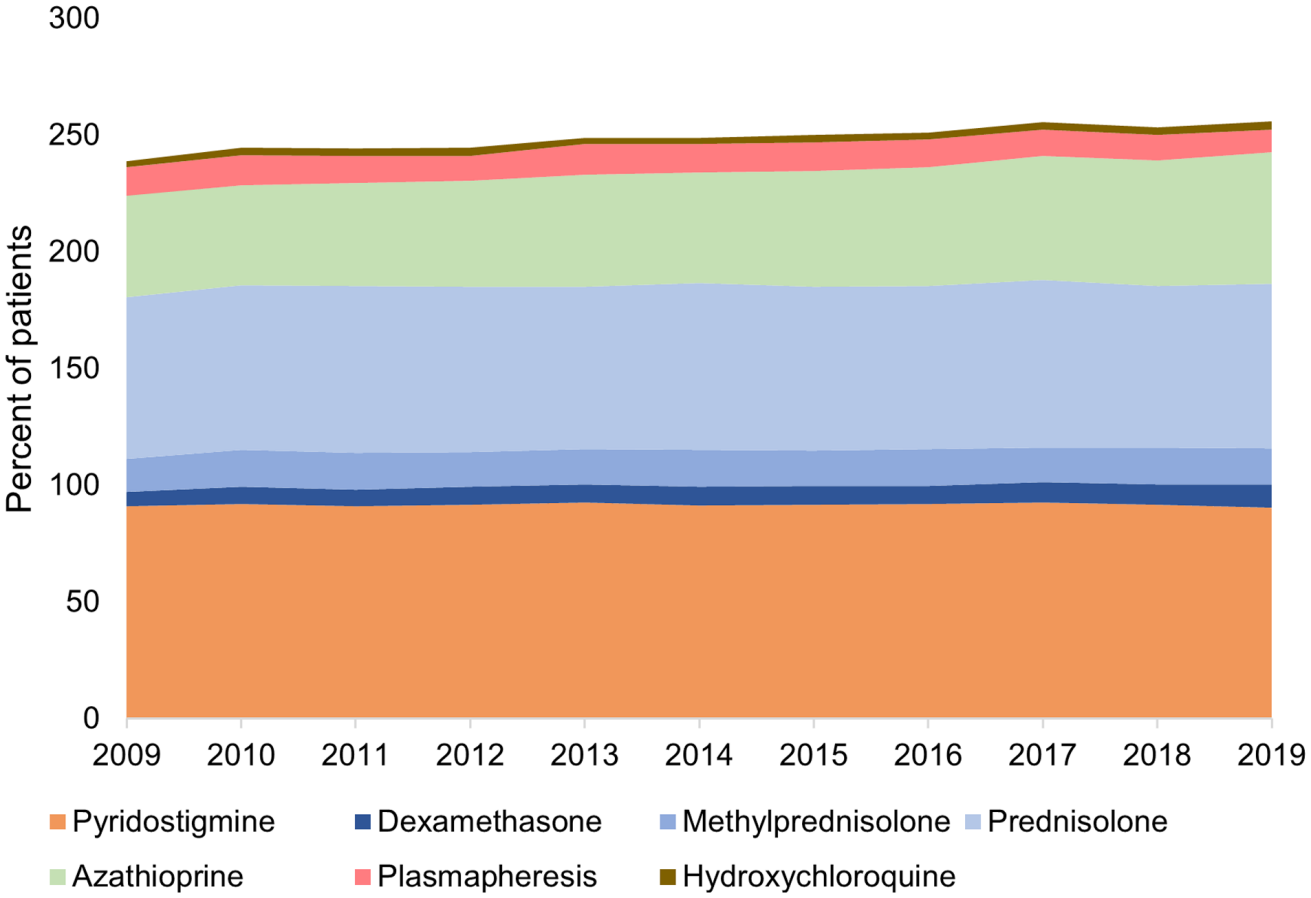
Treatments* used by patients for generalized myasthenia gravis in Taiwan, 2009–2019 *Treatments used by <5% of patients were excluded. The total is more than 100% because many patients co-used more than one therapy.

Medication type and usage changed little of the study period (Figure 3). Pyridostigmine was prescribed for approximately 90% of patients with gMG annually, followed by steroids in approximately 85% (around 70% prednisolone and 15% methylprednisolone). The use of azathioprine increased from 44% of patients in 2009 to 56% in 2019. Approximately 10% of patients in any study year underwent plasmapheresis. Other treatments were used infrequently; cyclosporine was used by 1.4% of patients in 2009 decreasing to 0.61% in 2019; mycophenolate mofetil by ≤2% of patients; and rituximab, leflunomide, methotrexate, tacrolimus and intravenous immunoglobulins, were each used by ≤0.7% of patients in any year.

First line treatment was with pyridostigmine in 82% of patients, steroids in 58% and azathioprine in 11% (Table 2). Intravenous steroids were used as first line treatment in 26% of patients, and plasmapheresis by 9%. For each treatment, mean and median treatment durations were strikingly different, indicating that some patients stayed on treatment for prolonged periods, as evidenced by maximum durations of as long as 110 months (pyridostigmine and steroids). The median (mean) treatment durations were 2.8 months (7.5 months) for pyridostigmine and for steroids, and 3 months (8.6 months) for azathioprine.

**Table 2:** First and second-line treatments for patients with generalized myasthenia gravis in Taiwan, 2009–2019.

	First line treatment (*N* = 6,506)							Second line 2nd treatment (*N* = 2,242)						
	*n*	%	Duration (months)					*n*	%	Duration (months)				
			Mean	Median	SD	Range	IQR			Mean	Median	SD	Range	IQR
Injections															
Steroid	1,608	24.72	2.41	1	4.52	1–107	1	612	25.31	3.01	2	4.49	1–42	2
Rituximab	6	0.09	5.83	3.5	6.18	1–16	9	4	0.17	5.25		2.22		
Immunoglobulin	6	0.09	2.5	2	1.38	1–5	1	4	0.17	1.75		0.96		
Plasmapheresis	597	9.18	2.89	2	3.31	1–29	3	515	21.30	2.78	2	3.69	1–54	2
Oral drugs															
Pyridostigmine	5,309	81.60	7.53	2.8	11.46	0.03–110.3	8.2	2,316	95.78	11.8	6.53	13.66	0.03–95.9	13.55
Steroid	3,774	58.01	7.52	2.8	11.56	0.03–110.3	8.33	1989	82.26	10.92	6.07	13.42	0.03–114	12.33
Azathioprine	727	11.17	8.6	3	11.74	0.03–74.23	11.1	832	34.41	12.33	7.2	13.86	0.03–90.57	16.47
Hydroxychloroquine	123	1.89	6.34	0.8	11.99	0.03–58.53	5.33	106	4.38	7.52	3	10.25	0.03–65.4	10.27
Mycophenolate mofetil/Mycophenolic acid	18	0.28	15.33	6.17	18.61	0.03–54.13	27.16	30	1.24	12.31	7.73	18.6	0.47–95.9	9.16
Methotrexate	15	0.23	8.33	0.93	12.07	0.03–35.67	22.34	13	0.54	8.26	5.6	8.78	0.1–26.7	13.6
Cyclosporine	9	0.14	3.56	1.1	7.48	0.03–23.33	1.7	13	0.54	10.75	3.73	15.13	0.7–51.1	8.33

IQR, inter-quartile range; SD, standard deviation.

A total of 34.5% (2,242/6506) of patients received second line treatment during the study period. The most frequently used second line treatments were pyridostigmine (96%), steroids (82%), azathioprine (34%), plasmapheresis (21%) and intravenous steroids (25%; Table 2). As for first line therapy, some patients remained on second line for an extended period, up to 114 months for steroids. The median (mean) treatment durations for second line drugs were 7 months (12 months) for pyridostigmine, 6 months (11 months) for steroids, and 7.2 months (12 months) for azathioprine.

Few patients (<2%) used methotrexate, mycophenolate mofetil, rituximab, cyclosporine, or intravenous immunoglobulin for either first or second line treatment.

### 3.3. HCV and HBV reactivation

The percentage of prevalent patients with HCV infection during each study year ranged from 2.01 to 4.35% annually with no evidence of a temporal tend.

There were 147 new HBV infections during the study, of which 32 (22%) received at least 4 weeks of antiviral therapy suggesting chronic infection. Among 334 patients who had a prior history of HBV infection, 24 (7.2%) developed HBV reactivation (defined as re-commencing antivirals treatment for a diagnosis of HBV infection after discontinuation of treatment for at least 6 months) over the study period.

## 4. Discussion

Using the population-based NHIRD we observed that the epidemiology of gMG is evolving in Taiwan. We show a significantly increasing prevalence rate over time that occurs from age 30 years in women and age 40 years in men. The mean age of prevalent cases increased from 52 to 55 years between 2009 and 2019. In 2019, the annual prevalence of gMG was 11.18 per 100,000 population and the incidence rate was 2.95 per 100,000 population. The increasing prevalence of gMG alongside stable incidence and all-cause fatality is likely to be a function of the aging population and the availability of improved treatments, with more patients with gMG surviving into older age.

Our results complement and build on a previous study using the same database conducted by Lai et al. (14), using data between 2000 and 2007. Lai et al. included all cases of MG (both ocular and gMG) as diagnosed by ICD9 codes and estimated a prevalence rate in 2007 of 14.0 per 100,000 population, increasing from 8.4 per 100,000 population in 2000. There was no evidence for any temporal trends in incidence rates (14). Unlike Lai et al., we observed an increase in incidence and prevalence rates in women at 30–39 years of age compared to men, and a higher incidence in males than females in older age; a disease pattern prevalent in some Caucasian populations and in Japan but observed less frequently in other Asian populations (1, 6, 14, 15). Although the results of the two studies cannot be directly compared because of the different definitions of MG used, they demonstrate a consistent trend of a stable incidence of MG/gMG in Taiwan, but a steady and continuing increase in prevalence, with a case shift to older populations. Both studies suggest a growing burden of disease and a growing impact on related healthcare costs.

As expected, a majority of patients received treatment with pyridostigmine and/or steroids, which is consistent with a real-world study in Korea (16), although steroid and/or azathioprine use were among the components of the gMG definition, and thus the prevalence of these treatments may be increased. Very few patients used immunosuppressants other than azathioprine in Taiwan, whereas 7% used cyclosporine, 6% used mycophenolate mofetil and 3% used tacrolimus in Korea (16). The infrequent use of other treatments reflects reimbursement policy under Taiwan’s health insurance. IVIG is only reimbursed in Taiwan for the management of myasthenia crisis, mycophenolate mofetil is not re-imbursed for used in MG, rituximab is usually reserved for severe cases or following crisis management, and methotrexate and cyclosporine are usually prescribed when patients have an insufficient response to azathioprine.

The annual prevalence of HCV infection in patients with gMG was within the range reported for the general population of Taiwan; 2.1% (95% CI 1.0–3.7; 2015) (17), suggesting no increased risk of HCV in patients with gMG.

HBV reactivation is associated with chronic immune suppression, frequently a result of chemotherapy, chronic high-dose steroid use, malignancy, and some biologics such as rituximab (18). Rates of HBV reactivation are highly variable and influenced by age, gender, the infecting HBV genotype, and the HBV disease phase (18); for example, the risk of reactivation in HBV carriers with lymphoid malignancies who are receiving rituximab can be >30% (19). HBV reactivation was reported in 11.1% of Asian patients with asthma or chronic respiratory disease treated with systemic steroids (20). In our study, the HBV reactivation rate in patients with gMG and prior history of HBV was 7.2%. We also observed that 22% of patients who developed HBV during the study used antivirals for at least 4 weeks, potentially suggesting a transition to chronic infection (or reactivation). This is higher than the reported chronicity rate of approximately 5% in adults (21). These results are difficult to interpret given the absence of clinical information available in the NHIRD, such as HBV disease phase, and HBV e antigen and HBsAg status. Moreover, in view of the small number of patients, we did not make links between HBV status and treatment regimens. It is worth noting however that prophylactic antivirals are not reimbursed by Taiwan’s health insurance and are therefore are not captured in the claims-based NHIRD. Newly prescribed antiviral treatment therefore indicates reactivation. Despite the limitations of these results, the data suggest a not negligible occurrence of HBV infection and HBV reactivation in patients with gMG, perhaps as a result of prolonged steroid use - steroids were used by at least 85% of patients during their treatment journey. We found no previously published information about the risk of HBV reactivation in patients with MG. However, our data suggest that some patients with gMG may be at risk of HBV infection or reactivation, and that patients considering long term steroid treatment might benefit from HBV screening prior to commencing treatment. Patients known to be HBV positive should be monitored regularly and antiviral prophylaxis might be considered in patients with underlying factors that puts them at higher risk of reactivation during steroid treatment.

Strengths of the study include comprehensive data capture by the NHIRD across the whole population of Taiwan, regardless of the location of the service provided, and the use of a 10-year study period that allowed us to assess temporal trends. In contrast to many studies of MG epidemiology that have assessed the overall syndrome of MG, we attempted to identify the more severe entity of gMG based on a history of hospitalization and/or receiving relevant treatment. Our definition may have excluded patients with ocular MG but is more useful for health technology assessments for new treatments which focus on patients with the most severe disease.

Limitations of the study include the lack of clinical information available in the database to determine disease severity, including distinguishing between ocular MG and gMG, response to treatment, or the rationale underlying treatment decision making. Incidence rates in our study should be interpreted cautiously because there is no specific ICD code for gMG, which necessitated the use of proxy criteria such as the use of relevant treatments or a specific number of visits. Other potential limitations that apply to all studies that use data from claims-based databases include coding accuracy, completeness, and data quality. However, data quality in the NHIRD is high, and demographic information, date and type of all health services provided is verified by the National Health Insurance Administration (22, 23). Finally, due to the absence of laboratory results such as liver enzymes or viral load in the database, we were unable to confirm whether patients who commenced anti-viral treatment for HBV had new infection or reactivation of disease.

The epidemiology of gMG in Taiwan is evolving rapidly, with higher prevalence rates and increasing numbers of older persons affected by the disease. The data suggest that the burden of disease and associated healthcare costs due to gMG are increasing. HBV infection or reactivation may pose a previously under-appreciated risk for patients with gMG receiving immunosuppressants, particularly in countries such as Taiwan where HBV is prevalent (11). Further assessment of this risk and consideration of measures to reduce the risk of HBV infection/reactivation in patients with gMG is needed.

## Data availability statement

The data analyzed in this study was obtained from the Taiwan’s National Health Insurance Research Database (NHIRD), which is provided by the National Health Insurance Administration (NHIA) and maintained by the Health and Welfare Data Science Center (HWDC; https://dep.mohw.gov.tw/DOS/cp-5119-59201-113.html), Ministry of Health and Welfare, Executive Yuan, Taiwan. The following licenses/restrictions apply: the Taiwanese government prohibits the release of the NHI claims dataset to the public domain. Interested researchers can obtain the data through formal application to the HWDC. Requests to access these datasets should be directed to the HWDC, https://dep.mohw.gov.tw/DOS/cp-5119-59201-113.html.

## Ethics statement

Ethical review and approval was not required for the study on human participants in accordance with the local legislation and institutional requirements. Written informed consent from the patients/participants or patients/participants’ legal guardian/next of kin was not required to participate in this study in accordance with the national legislation and the institutional requirements.

## Author contributions

S-PS: software, formal analysis, data curation, visualization, writing – review, and editing. KH: conceptualization, project administration, writing – review, and editing. YL: conceptualization, methodology, supervision, writing – review, and editing. C-CY: methodology, writing – review, and editing. C-HT: study conceptualization, methodology, data curation, formal analysis, writing – review, and editing. All authors contributed to the article and approved the submitted version.

## Funding

This work was supported by Janssen Asia Pacific, a division of Johnson and Johnson Pte Ltd.

## Conflict of interest

KH is an employee of Janssen Asia Pacific. YL is an employee of Epidemiology, Office of the Chief Medical Officer, Johnson and Johnson. KH and YL hold stock in Johnson & Johnson.

The authors declare that this study received funding from Janssen Asia Pacific, a division of Johnson and Johnson Pte Ltd. The funder was involved in the study design and provided medical writing support. The funder was not involved in collection, analysis, interpretation of data or the decision to submit it for publication.

## Publisher’s note

All claims expressed in this article are solely those of the authors and do not necessarily represent those of their affiliated organizations, or those of the publisher, the editors and the reviewers. Any product that may be evaluated in this article, or claim that may be made by its manufacturer, is not guaranteed or endorsed by the publisher.
