# Supplementary material for: The growing burden of generalized myasthenia gravis: a population-based retrospective cohort study in Taiwan
Source: Front Neurol. 2023 Jun 23;14:1203679. doi: 10.3389/fneur.2023.1203679 (PMC10327564; doi:10.3389/fneur.2023.1203679)
Supplement: Supplementary file 1 [file Data_Sheet_1.docx]

Supplementary Material

The growing burden of generalized myasthenia gravis: a population-based retrospective cohort study in Taiwan

Keira Joann Herr, Shih-Pei Shen, Yanfang Liu, Chih-Chao Yang, Chao-Hsiun Tang

*** Correspondence: Chih-Chao Yang. Email** [**jesse6627@gmail.com**](mailto:jesse6627@gmail.com)

# Supplementary Figures and Tables

## Supplementary Tables

**Table S1** Annual prevalence rate (per 100,000 population) of any myasthenia gravis by calendar year, age, and gender

|  | **Number of persons with myasthenia gravis** | | | | **Prevalence rate** | | |
| --- | --- | --- | --- | --- | --- | --- | --- |
|  | **Male** | **Female** | **Missing** | **Total** | **Male** | **Female** | **Total** |
| **2009** | 1472 | 2161 | 14 | 3647 | 12.66 | 18.88 | 15.80 |
| **2010** | 1629 | 2335 | 13 | 3977 | 14.00 | 20.30 | 17.19 |
| **2011** | 1706 | 2444 | 13 | 4163 | 14.66 | 21.15 | 17.95 |
| **2012** | 1810 | 2551 | 18 | 4379 | 15.52 | 21.97 | 18.82 |
| **2013** | 1886 | 2703 | 15 | 4604 | 16.15 | 23.17 | 19.72 |
| **2014** | 2042 | 2853 | 20 | 4915 | 17.47 | 24.36 | 21.00 |
| **2015** | 2155 | 2986 | 17 | 5158 | 18.41 | 25.40 | 21.98 |
| **2016** | 2254 | 3132 | 19 | 5405 | 19.24 | 26.54 | 22.98 |
| **2017** | 2349 | 3250 | 19 | 5618 | 20.04 | 27.46 | 23.85 |
| **2018** | 2523 | 3339 | 24 | 5886 | 21.53 | 28.14 | 24.96 |
| **2019** | 2710 | 3554 | 29 | 6293 | 23.14 | 29.90 | 26.67 |

**Table S2**  Annual prevalence of gMG by calendar year, age and gender in Taiwan per 100,000 population

|  | **2009** | | | | | | | | | | | | | | |
| --- | --- | --- | --- | --- | --- | --- | --- | --- | --- | --- | --- | --- | --- | --- | --- |
|  | **Male** | | | | | **Female** | | | | | **Total** | | | | |
| **Age** | **No of gMG** | **Total persons (mid-year)** | **Prevalence rate** | **95% CI** | | **No of gMG** | **Total persons (mid-year)** | **Prevalence rate** | **95% CI** | | **No of gMG** | **Total persons (mid-year)** | **Prevalence rate** | **95% CI** | |
|  |  |  |  | **LL** | **UL** |  |  |  | **LL** | **UL** |  |  |  | **LL** | **UL** |
| ≤ 9 | 12 | 1,202,532 | 1.00 | 0.52 | 1.74 | 8 | 1,099,550 | 0.73 | 0.31 | 1.43 | 20 | 2,302,082 | 0.87 | 0.53 | 1.34 |
| 10 – 19 | 9 | 1,641,301 | 0.55 | 0.25 | 1.04 | 19 | 1,510,368 | 1.26 | 0.76 | 1.96 | 28 | 3,151,669 | 0.89 | 0.59 | 1.28 |
| 20 – 29 | 35 | 1,829,403 | 1.91 | 1.33 | 2.66 | 79 | 1,759,416 | 4.49 | 3.55 | 5.60 | 117 | 3,588,819 | 3.26 | 2.70 | 3.91 |
| 30 – 39 | 78 | 1,882,156 | 4.14 | 3.28 | 5.17 | 139 | 1,881,517 | 7.39 | 6.21 | 8.72 | 220 | 3,763,673 | 5.85 | 5.10 | 6.67 |
| 40 – 49 | 146 | 1,894,307 | 7.71 | 6.51 | 9.06 | 193 | 1,879,668 | 10.27 | 8.87 | 11.82 | 339 | 3,773,975 | 8.98 | 8.05 | 9.99 |
| 50 – 59 | 147 | 1,582,351 | 9.29 | 7.85 | 10.92 | 180 | 1,613,496 | 11.16 | 9.59 | 12.91 | 328 | 3,195,847 | 10.26 | 9.18 | 11.44 |
| 60 – 69 | 113 | 788,841 | 14.32 | 11.81 | 17.22 | 134 | 846,859 | 15.82 | 13.26 | 18.74 | 247 | 1,635,700 | 15.10 | 13.28 | 17.11 |
| 70 – 79 | 107 | 532,440 | 20.10 | 16.47 | 24.28 | 94 | 581,552 | 16.16 | 13.06 | 19.78 | 201 | 1,113,992 | 18.04 | 15.64 | 20.72 |
| ≥ 80 | 35 | 278,212 | 12.58 | 8.76 | 17.50 | 41 | 274,433 | 14.94 | 10.72 | 20.27 | 76 | 552,645 | 13.75 | 10.84 | 17.21 |
| **Total** | 682 | 11,631,543 | 5.86 | 5.43 | 6.32 | 887 | 11,446,859 | 7.75 | 7.25 | 8.28 | 1576 | 23,078,402 | 6.83 | 6.50 | 7.17 |

|  | **2010** | | | | | | | | | | | | | | |
| --- | --- | --- | --- | --- | --- | --- | --- | --- | --- | --- | --- | --- | --- | --- | --- |
|  | **Male** | | | | | **Female** | | | | | **Total** | | | | |
| **Age** | **No of gMG** | **Total persons (mid-year)** | **Prevalence rate** | **95% CI** | | **No of gMG** | **Total persons (mid-year)** | **Prevalence rate** | **95% CI** | | **No of gMG** | **Total persons (mid-year)** | **Prevalence rate** | **95% CI** | |
|  |  |  |  | **LL** | **UL** |  |  |  | **LL** | **UL** |  |  |  | **LL** | **UL** |
| ≤ 9 | 7 | 1,143,989 | 0.61 | 0.25 | 1.26 | 12 | 1,046,252 | 1.15 | 0.59 | 2.00 | 19 | 2,190,241 | 0.87 | 0.52 | 1.35 |
| 10 – 19 | 9 | 1,627,004 | 0.55 | 0.25 | 1.05 | 14 | 1,497,160 | 0.94 | 0.51 | 1.57 | 23 | 3,124,164 | 0.74 | 0.47 | 1.10 |
| 20 – 29 | 40 | 1,789,374 | 2.24 | 1.60 | 3.04 | 76 | 1,717,728 | 4.42 | 3.49 | 5.54 | 118 | 3,507,102 | 3.36 | 2.78 | 4.03 |
| 30 – 39 | 78 | 1,895,705 | 4.11 | 3.25 | 5.14 | 158 | 1,908,246 | 8.28 | 7.04 | 9.68 | 237 | 3,803,951 | 6.23 | 5.46 | 7.08 |
| 40 – 49 | 136 | 1,888,270 | 7.20 | 6.04 | 8.52 | 197 | 1,882,181 | 10.47 | 9.06 | 12.03 | 336 | 3,770,451 | 8.91 | 7.98 | 9.92 |
| 50 – 59 | 167 | 1,633,507 | 10.22 | 8.73 | 11.90 | 203 | 1,669,740 | 12.16 | 10.54 | 13.95 | 372 | 3,303,247 | 11.26 | 10.15 | 12.47 |
| 60 – 69 | 128 | 830,321 | 15.42 | 12.86 | 18.33 | 146 | 889,164 | 16.42 | 13.86 | 19.31 | 274 | 1,719,485 | 15.94 | 14.10 | 17.94 |
| 70 – 79 | 117 | 532,608 | 21.97 | 18.17 | 26.33 | 108 | 602,213 | 17.93 | 14.71 | 21.65 | 225 | 1,134,821 | 19.83 | 17.32 | 22.59 |
| ≥ 80 | 41 | 295,202 | 13.89 | 9.97 | 18.84 | 52 | 292,284 | 17.79 | 13.29 | 23.33 | 93 | 587,486 | 15.83 | 12.78 | 19.39 |
| **Total** | 723 | 1,635,980 | 6.21 | 5.77 | 6.68 | 966 | 1,504,968 | 8.40 | 7.88 | 8.94 | 1697 | 23,140,948 | 7.33 | 6.99 | 7.69 |

|  | **2011** | | | | | | | | | | | | | | |
| --- | --- | --- | --- | --- | --- | --- | --- | --- | --- | --- | --- | --- | --- | --- | --- |
|  | **Male** | | | | | **Female** | | | | | **Total** | | | | |
| **Age** | **No of gMG** | **Total persons (mid-year)** | **Prevalence rate** | **95% CI** | | **No of gMG** | **Total persons (mid-year)** | **Prevalence rate** | **95% CI** | | **No of gMG** | **Total persons (mid-year)** | **Prevalence rate** | **95% CI** | |
|  |  |  |  | **LL** | **UL** |  |  |  | **LL** | **UL** |  |  |  | **LL** | **UL** |
| ≤ 9 | 5 | 1,093,578 | 0.46 | 0.15 | 1.07 | 9 | 1,000,309 | 0.90 | 0.41 | 1.71 | 14 | 2,093,887 | 0.67 | 0.37 | 1.12 |
| 10 – 19 | 11 | 1,602,918 | 0.69 | 0.34 | 1.23 | 17 | 1,476,441 | 1.15 | 0.67 | 1.84 | 28 | 3,079,359 | 0.91 | 0.60 | 1.31 |
| 20 – 29 | 36 | 1,753,636 | 2.05 | 1.44 | 2.84 | 70 | 1,674,503 | 4.18 | 3.26 | 5.28 | 107 | 3,428,139 | 3.12 | 2.56 | 3.77 |
| 30 – 39 | 82 | 1,911,975 | 4.29 | 3.41 | 5.32 | 177 | 1,932,260 | 9.16 | 7.86 | 10.61 | 262 | 3,844,235 | 6.82 | 6.02 | 7.69 |
| 40 – 49 | 140 | 1,877,616 | 7.46 | 6.27 | 8.80 | 195 | 1,879,939 | 10.37 | 8.97 | 11.94 | 335 | 3,757,555 | 8.92 | 7.99 | 9.92 |
| 50 – 59 | 191 | 1,670,830 | 11.43 | 9.87 | 13.17 | 211 | 1,710,141 | 12.34 | 10.73 | 14.12 | 402 | 3,380,971 | 11.89 | 10.76 | 13.11 |
| 60 – 69 | 143 | 884,889 | 16.16 | 13.62 | 19.04 | 143 | 947,106 | 15.10 | 12.73 | 17.79 | 287 | 1,831,995 | 15.67 | 13.91 | 17.59 |
| 70 – 79 | 113 | 535,632 | 21.10 | 17.39 | 25.36 | 137 | 621,881 | 22.03 | 18.50 | 26.04 | 250 | 1,157,513 | 21.60 | 19.00 | 24.45 |
| ≥ 80 | 55 | 309,376 | 17.78 | 13.39 | 23.14 | 53 | 310,488 | 17.07 | 12.79 | 22.33 | 108 | 619,864 | 17.42 | 14.29 | 21.04 |
| **Total** | 776 | 1,640,450 | 6.67 | 6.21 | 7.15 | 1012 | 1,553,068 | 8.76 | 8.23 | 9.32 | 1793 | 23,193,518 | 7.73 | 7.38 | 8.10 |

|  | **2012** | | | | | | | | | | | | | | |
| --- | --- | --- | --- | --- | --- | --- | --- | --- | --- | --- | --- | --- | --- | --- | --- |
|  | **Male** | | | | | **Female** | | | | | **Total** | | | | |
| **Age** | **No of gMG** | **Total persons (mid-year)** | **Prevalence rate** | **95% CI** | | **No of gMG** | **Total persons (mid-year)** | **Prevalence rate** | **95% CI** | | **No of gMG** | **Total persons (mid-year)** | **Prevalence rate** | **95% CI** | |
|  |  |  |  | **LL** | **UL** |  |  |  | **LL** | **UL** |  |  |  | **LL** | **UL** |
| ≤ 9 | 7 | 1,074,447 | 0.65 | 0.26 | 1.34 | 6 | 984,220 | 0.61 | 0.22 | 1.33 | 13 | 2,058,667 | 0.63 | 0.34 | 1.08 |
| 10 – 19 | 11 | 1,567,316 | 0.70 | 0.35 | 1.26 | 13 | 1,444,632 | 0.90 | 0.48 | 1.54 | 24 | 3,011,948 | 0.80 | 0.51 | 1.19 |
| 20 – 29 | 35 | 1,715,440 | 2.04 | 1.42 | 2.84 | 62 | 1,627,987 | 3.81 | 2.92 | 4.88 | 100 | 3,343,427 | 2.99 | 2.43 | 3.64 |
| 30 – 39 | 76 | 1,933,325 | 3.93 | 3.10 | 4.92 | 183 | 1,957,192 | 9.35 | 8.04 | 10.81 | 262 | 3,890,517 | 6.73 | 5.94 | 7.60 |
| 40 – 49 | 135 | 1,859,413 | 7.26 | 6.09 | 8.59 | 192 | 1,870,565 | 10.26 | 8.86 | 11.82 | 328 | 3,729,978 | 8.79 | 7.87 | 9.80 |
| 50 – 59 | 192 | 1,699,874 | 11.29 | 9.75 | 13.01 | 219 | 1,741,936 | 12.57 | 10.96 | 14.35 | 411 | 3,441,810 | 11.94 | 10.81 | 13.15 |
| 60 – 69 | 138 | 949,386 | 14.54 | 12.21 | 17.17 | 135 | 1,016,443 | 13.28 | 11.14 | 15.72 | 275 | 1,965,829 | 13.99 | 12.38 | 15.74 |
| 70 – 79 | 114 | 541,220 | 21.06 | 17.38 | 25.30 | 126 | 639,615 | 19.70 | 16.41 | 23.45 | 240 | 1,180,835 | 20.32 | 17.83 | 23.06 |
| ≥ 80 | 63 | 319,076 | 19.74 | 15.17 | 25.26 | 58 | 328,280 | 17.67 | 13.42 | 22.84 | 121 | 647,356 | 18.69 | 15.51 | 22.33 |
| **Total** | 771 | 1,659,497 | 6.61 | 6.15 | 7.10 | 994 | 1,610,870 | 8.56 | 8.04 | 9.11 | 1774 | 23,270,367 | 7.62 | 7.27 | 7.99 |

|  | **2013** | | | | | | | | | | | | | | |
| --- | --- | --- | --- | --- | --- | --- | --- | --- | --- | --- | --- | --- | --- | --- | --- |
|  | **Male** | | | | | **Female** | | | | | **Total** | | | | |
| **Age** | **No of gMG** | **Total persons (mid-year)** | **Prevalence rate** | **95% CI** | | **No of gMG** | **Total persons (mid-year)** | **Prevalence rate** | **95% CI** | | **No of gMG** | **Total persons (mid-year)** | **Prevalence rate** | **95% CI** | |
|  |  |  |  | **LL** | **UL** |  |  |  | **LL** | **UL** |  |  |  | **LL** | **UL** |
| ≤ 9 | 5 | 1,063,150 | 0.47 | 0.15 | 1.10 | 5 | 976,290 | 0.51 | 0.17 | 1.20 | 10 | 2,039,440 | 0.49 | 0.24 | 0.90 |
| 10 – 19 | 10 | 1,523,253 | 0.66 | 0.31 | 1.21 | 21 | 1,402,684 | 1.50 | 0.93 | 2.29 | 31 | 2,925,937 | 1.06 | 0.72 | 1.50 |
| 20 – 29 | 28 | 1,686,667 | 1.66 | 1.10 | 2.40 | 61 | 1,591,357 | 3.83 | 2.93 | 4.92 | 89 | 3,278,024 | 2.72 | 2.18 | 3.34 |
| 30 – 39 | 96 | 1,950,014 | 4.92 | 3.99 | 6.01 | 190 | 1,975,810 | 9.62 | 8.30 | 11.09 | 291 | 3,925,824 | 7.41 | 6.59 | 8.31 |
| 40 – 49 | 146 | 1,836,680 | 7.95 | 6.71 | 9.35 | 206 | 1,855,392 | 11.10 | 9.64 | 12.73 | 354 | 3,692,072 | 9.59 | 8.62 | 10.64 |
| 50 – 59 | 188 | 1,730,735 | 10.86 | 9.37 | 12.53 | 237 | 1,776,225 | 13.34 | 11.70 | 15.15 | 425 | 3,506,960 | 12.12 | 10.99 | 13.33 |
| 60 – 69 | 148 | 1,013,358 | 14.60 | 12.35 | 17.16 | 178 | 1,085,597 | 16.40 | 14.08 | 18.99 | 328 | 2,098,955 | 15.63 | 13.98 | 17.41 |
| 70 – 79 | 131 | 549,782 | 23.83 | 19.92 | 28.27 | 142 | 656,412 | 21.63 | 18.22 | 25.50 | 273 | 1,206,194 | 22.63 | 20.03 | 25.48 |
| ≥ 80 | 72 | 325,358 | 22.13 | 17.32 | 27.87 | 51 | 345,906 | 14.74 | 10.98 | 19.39 | 123 | 671,264 | 18.32 | 15.23 | 21.86 |
| **Total** | 824 | 11,678,997 | 7.06 | 6.58 | 7.55 | 1091 | 1,665,673 | 9.35 | 8.81 | 9.92 | 1924 | 23,344,670 | 8.24 | 7.88 | 8.62 |

|  | **2014** | | | | | | | | | | | | | | |
| --- | --- | --- | --- | --- | --- | --- | --- | --- | --- | --- | --- | --- | --- | --- | --- |
|  | **Male** | | | | | **Female** | | | | | **Total** | | | | |
| **Age** | **No of gMG** | **Total persons (mid-year)** | **Prevalence rate** | **95% CI** | | **No of gMG** | **Total persons (mid-year)** | **Prevalence rate** | **95% CI** | | **No of gMG** | **Total persons (mid-year)** | **Prevalence rate** | **95% CI** | |
|  |  |  |  | **LL** | **UL** |  |  |  | **LL** | **UL** |  |  |  | **LL** | **UL** |
| ≤ 9 | 6 | 1,053,756 | 0.57 | 0.21 | 1.24 | 9 | 970,675 | 0.93 | 0.42 | 1.76 | 15 | 2,024,431 | 0.74 | 0.41 | 1.22 |
| 10 – 19 | 10 | 1,471,907 | 0.68 | 0.33 | 1.25 | 24 | 1,352,990 | 1.77 | 1.14 | 2.64 | 34 | 2,824,897 | 1.20 | 0.83 | 1.68 |
| 20 – 29 | 36 | 1,665,910 | 2.16 | 1.51 | 2.99 | 80 | 1,563,666 | 5.12 | 4.06 | 6.37 | 117 | 3,229,576 | 3.62 | 3.00 | 4.34 |
| 30 – 39 | 101 | 1,959,045 | 5.16 | 4.20 | 6.26 | 210 | 1,984,951 | 10.58 | 9.20 | 12.11 | 316 | 3,943,996 | 8.01 | 7.15 | 8.95 |
| 40 – 49 | 144 | 1,814,924 | 7.93 | 6.69 | 9.34 | 209 | 1,840,949 | 11.35 | 9.87 | 13.00 | 357 | 3,655,873 | 9.77 | 8.78 | 10.83 |
| 50 – 59 | 228 | 1,757,270 | 12.97 | 11.35 | 14.77 | 240 | 1,805,384 | 13.29 | 11.66 | 15.09 | 468 | 3,562,654 | 13.14 | 11.97 | 14.38 |
| 60 – 69 | 205 | 1,080,343 | 18.98 | 16.47 | 21.76 | 190 | 1,159,340 | 16.39 | 14.14 | 18.89 | 396 | 2,239,683 | 17.68 | 15.98 | 19.51 |
| 70 – 79 | 125 | 559,088 | 22.36 | 18.61 | 26.64 | 136 | 670,277 | 20.29 | 17.02 | 24.00 | 261 | 1,229,365 | 21.23 | 18.73 | 23.97 |
| ≥ 80 | 68 | 329,080 | 20.66 | 16.05 | 26.20 | 69 | 364,080 | 18.95 | 14.75 | 23.98 | 137 | 693,160 | 19.76 | 16.59 | 23.36 |
| **Total** | 923 | 11,691,323 | 7.89 | 7.39 | 8.42 | 1167 | 11,712,312 | 9.96 | 9.40 | 10.55 | 2101 | 23,403,635 | 8.98 | 8.60 | 9.37 |

|  | **2015** | | | | | | | | | | | | | | |
| --- | --- | --- | --- | --- | --- | --- | --- | --- | --- | --- | --- | --- | --- | --- | --- |
|  | **Male** | | | | | **Female** | | | | | **Total** | | | | |
| **Age** | **No of gMG** | **Total persons (mid-year)** | **Prevalence rate** | **95% CI** | | **No of gMG** | **Total persons (mid-year)** | **Prevalence rate** | **95% CI** | | **No of gMG** | **Total persons (mid-year)** | **Prevalence rate** | **95% CI** | |
|  |  |  |  | **LL** | **UL** |  |  |  | **LL** | **UL** |  |  |  | **LL** | **UL** |
| ≤ 9 | 9 | 1,053,497 | 0.85 | 0.39 | 1.62 | 6 | 972,459 | 0.62 | 0.23 | 1.34 | 15 | 2,025,956 | 0.74 | 0.41 | 1.22 |
| 10 – 19 | 10 | 1,415,840 | 0.71 | 0.34 | 1.30 | 20 | 1,299,562 | 1.54 | 0.94 | 2.38 | 30 | 2,715,402 | 1.10 | 0.75 | 1.58 |
| 20 – 29 | 34 | 1,654,003 | 2.06 | 1.42 | 2.87 | 59 | 1,544,753 | 3.82 | 2.91 | 4.93 | 93 | 3,198,756 | 2.91 | 2.35 | 3.56 |
| 30 – 39 | 94 | 1,957,524 | 4.80 | 3.88 | 5.88 | 209 | 1,980,834 | 10.55 | 9.17 | 12.08 | 305 | 3,938,358 | 7.74 | 6.90 | 8.66 |
| 40 – 49 | 137 | 1,797,820 | 7.62 | 6.40 | 9.01 | 234 | 1,832,139 | 12.77 | 11.19 | 14.52 | 373 | 3,629,959 | 10.28 | 9.26 | 11.37 |
| 50 – 59 | 229 | 1,773,162 | 12.91 | 11.30 | 14.70 | 259 | 1,824,163 | 14.20 | 12.52 | 16.04 | 488 | 3,597,325 | 13.57 | 12.39 | 14.82 |
| 60 – 69 | 231 | 1,159,208 | 19.93 | 17.44 | 22.67 | 218 | 1,245,763 | 17.50 | 15.25 | 19.98 | 450 | 2,404,971 | 18.71 | 17.02 | 20.52 |
| 70 – 79 | 113 | 561,957 | 20.11 | 16.57 | 24.18 | 145 | 675,113 | 21.48 | 18.12 | 25.27 | 259 | 1,237,070 | 20.94 | 18.46 | 23.65 |
| ≥ 80 | 64 | 331,998 | 19.28 | 14.85 | 24.62 | 75 | 383,119 | 19.58 | 15.40 | 24.54 | 139 | 715,117 | 19.44 | 16.34 | 22.95 |
| **Total** | 921 | 1,705,009 | 7.87 | 7.37 | 8.39 | 1225 | 11,757,905 | 10.42 | 9.84 | 11.02 | 2152 | 23,462,914 | 9.17 | 8.79 | 9.57 |

|  | **2016** | | | | | | | | | | | | | | |
| --- | --- | --- | --- | --- | --- | --- | --- | --- | --- | --- | --- | --- | --- | --- | --- |
|  | **Male** | | | | | **Female** | | | | | **Total** | | | | |
| **Age** | **No of gMG** | **Total persons (mid-year)** | **Prevalence rate** | **95% CI** | | **No of gMG** | **Total persons (mid-year)** | **Prevalence rate** | **95% CI** | | **No of gMG** | **Total persons (mid-year)** | **Prevalence rate** | **95% CI** | |
|  |  |  |  | **LL** | **UL** |  |  |  | **LL** | **UL** |  |  |  | **LL** | **UL** |
| ≤ 9 | 9 | 1,056,087 | 0.85 | 0.39 | 1.62 | 5 | 976,064 | 0.51 | 0.17 | 1.20 | 14 | 2,032,151 | 0.69 | 0.38 | 1.16 |
| 10 – 19 | 10 | 1,356,401 | 0.74 | 0.35 | 1.36 | 25 | 1,243,584 | 2.01 | 1.30 | 2.97 | 35 | 2,599,985 | 1.35 | 0.94 | 1.87 |
| 20 – 29 | 32 | 1,657,980 | 1.93 | 1.32 | 2.72 | 59 | 1,542,308 | 3.83 | 2.91 | 4.93 | 91 | 3,200,288 | 2.84 | 2.29 | 3.49 |
| 30 – 39 | 99 | 1,924,375 | 5.14 | 4.18 | 6.26 | 213 | 1,944,493 | 10.95 | 9.53 | 12.53 | 314 | 3,868,868 | 8.12 | 7.24 | 9.07 |
| 40 – 49 | 136 | 1,797,783 | 7.56 | 6.35 | 8.95 | 240 | 1,839,170 | 13.05 | 11.45 | 14.81 | 378 | 3,636,953 | 10.39 | 9.37 | 11.50 |
| 50 – 59 | 243 | 1,781,772 | 13.64 | 11.98 | 15.46 | 282 | 1,836,712 | 15.35 | 13.61 | 17.25 | 527 | 3,618,484 | 14.56 | 13.35 | 15.86 |
| 60 – 69 | 238 | 1,244,742 | 19.12 | 16.77 | 21.71 | 231 | 1,339,750 | 17.24 | 15.09 | 19.61 | 471 | 2,584,492 | 18.22 | 16.62 | 19.95 |
| 70 – 79 | 126 | 562,531 | 22.40 | 18.66 | 26.67 | 146 | 676,889 | 21.57 | 18.21 | 25.36 | 273 | 1,239,420 | 22.03 | 19.49 | 24.80 |
| ≥ 80 | 56 | 333,988 | 16.77 | 12.67 | 21.77 | 88 | 401,316 | 21.93 | 17.59 | 27.02 | 144 | 735,304 | 19.58 | 16.52 | 23.06 |
| **Total** | 949 | 1,715,659 | 8.10 | 7.59 | 8.63 | 1289 | 1,800,286 | 10.92 | 10.34 | 11.54 | 2247 | 23,515,945 | 9.56 | 9.16 | 9.96 |

|  | **2017** | | | | | | | | | | | | | | |
| --- | --- | --- | --- | --- | --- | --- | --- | --- | --- | --- | --- | --- | --- | --- | --- |
|  | **Male** | | | | | **Female** | | | | | **Total** | | | | |
| **Age** | **No of gMG** | **Total persons (mid-year)** | **Prevalence rate** | **95% CI** | | **No of gMG** | **Total persons (mid-year)** | **Prevalence rate** | **95% CI** | | **No of gMG** | **Total persons (mid-year)** | **Prevalence rate** | **95% CI** | |
|  |  |  |  | **LL** | **UL** |  |  |  | **LL** | **UL** |  |  |  | **LL** | **UL** |
| ≤ 9 | 4 | 1,053,816 | 0.38 | 0.10 | 0.97 | 6 | 975,764 | 0.61 | 0.23 | 1.34 | 10 | 2,029,580 | 0.49 | 0.24 | 0.91 |
| 10 – 19 | 9 | 1,296,290 | 0.69 | 0.32 | 1.32 | 26 | 1,187,431 | 2.19 | 1.43 | 3.21 | 35 | 2,483,721 | 1.41 | 0.98 | 1.96 |
| 20 – 29 | 38 | 1,668,512 | 2.28 | 1.61 | 3.13 | 71 | 1,548,458 | 4.59 | 3.58 | 5.78 | 109 | 3,216,970 | 3.39 | 2.78 | 4.09 |
| 30 – 39 | 97 | 1,879,059 | 5.16 | 4.19 | 6.30 | 181 | 1,892,798 | 9.56 | 8.22 | 11.06 | 281 | 3,771,857 | 7.45 | 6.60 | 8.37 |
| 40 – 49 | 144 | 1,808,930 | 7.96 | 6.71 | 9.37 | 259 | 1,858,061 | 13.94 | 12.29 | 15.74 | 405 | 3,666,991 | 11.04 | 9.99 | 12.17 |
| 50 – 59 | 244 | 1,786,986 | 13.65 | 11.99 | 15.48 | 288 | 1,844,418 | 15.61 | 13.86 | 17.53 | 532 | 3,631,404 | 14.65 | 13.43 | 15.95 |
| 60 – 69 | 270 | 1,316,924 | 20.50 | 18.13 | 23.10 | 256 | 1,421,204 | 18.01 | 15.87 | 20.36 | 526 | 2,738,128 | 19.21 | 17.60 | 20.92 |
| 70 – 79 | 146 | 572,847 | 25.49 | 21.52 | 29.97 | 147 | 688,478 | 21.35 | 18.04 | 25.09 | 293 | 1,261,325 | 23.23 | 20.65 | 26.05 |
| ≥ 80 | 71 | 336,061 | 21.13 | 16.50 | 26.65 | 88 | 419,485 | 20.98 | 16.83 | 25.84 | 159 | 755,546 | 21.04 | 17.90 | 24.58 |
| **Total** | 1023 | 1,719,425 | 8.73 | 8.20 | 9.28 | 1322 | 1,836,097 | 11.17 | 10.58 | 11.79 | 2350 | 23,555,522 | 9.98 | 9.58 | 10.39 |

|  | **2018** | | | | | | | | | | | | | | |
| --- | --- | --- | --- | --- | --- | --- | --- | --- | --- | --- | --- | --- | --- | --- | --- |
|  | **Male** | | | | | **Female** | | | | | **Total** | | | | |
| **Age** | **No of gMG** | **Total persons (mid-year)** | **Prevalence rate** | **95% CI** | | **No of gMG** | **Total persons (mid-year)** | **Prevalence rate** | **95% CI** | | **No of gMG** | **Total persons (mid-year)** | **Prevalence rate** | **95% CI** | |
|  |  |  |  | **LL** | **UL** |  |  |  | **LL** | **UL** |  |  |  | **LL** | **UL** |
| ≤ 9 | 4 | 1,046,336 | 0.38 | 0.10 | 0.98 | 8 | 970,859 | 0.82 | 0.36 | 1.62 | 12 | 2,017,195 | 0.59 | 0.31 | 1.04 |
| 10 – 19 | 17 | 1,248,418 | 1.36 | 0.79 | 2.18 | 31 | 1,142,606 | 2.71 | 1.84 | 3.85 | 48 | 2,391,024 | 2.01 | 1.48 | 2.66 |
| 20 – 29 | 38 | 1,653,946 | 2.30 | 1.63 | 3.15 | 77 | 1,533,181 | 5.02 | 3.96 | 6.28 | 116 | 3,187,127 | 3.64 | 3.01 | 4.37 |
| 30 – 39 | 101 | 1,848,096 | 5.47 | 4.45 | 6.64 | 186 | 1,851,811 | 10.04 | 8.65 | 11.60 | 293 | 3,699,907 | 7.92 | 7.04 | 8.88 |
| 40 – 49 | 167 | 1,818,995 | 9.18 | 7.84 | 10.68 | 259 | 1,875,770 | 13.81 | 12.18 | 15.60 | 428 | 3,694,765 | 11.58 | 10.51 | 12.74 |
| 50 – 59 | 243 | 1,789,423 | 13.58 | 11.93 | 15.40 | 297 | 1,849,071 | 16.06 | 14.29 | 18.00 | 540 | 3,638,494 | 14.84 | 13.62 | 16.15 |
| 60 – 69 | 308 | 1,376,599 | 22.37 | 19.95 | 25.02 | 277 | 1,489,570 | 18.60 | 16.47 | 20.92 | 587 | 2,866,169 | 20.48 | 18.86 | 22.21 |
| 70 – 79 | 153 | 594,860 | 25.72 | 21.81 | 30.13 | 170 | 712,093 | 23.87 | 20.42 | 27.74 | 323 | 1,306,953 | 24.71 | 22.09 | 27.56 |
| ≥ 80 | 78 | 339,574 | 22.97 | 18.16 | 28.67 | 95 | 438,872 | 21.65 | 17.51 | 26.46 | 174 | 778,446 | 22.35 | 19.15 | 25.93 |
| **Total** | 1109 | 1,716,247 | 9.47 | 8.92 | 10.04 | 1400 | 1,863,833 | 11.80 | 11.19 | 12.44 | 2521 | 23,580,080 | 10.69 | 10.28 | 11.12 |

|  | **2019** | | | | | | | | | | | | | | |
| --- | --- | --- | --- | --- | --- | --- | --- | --- | --- | --- | --- | --- | --- | --- | --- |
|  | **Male** | | | | | **Female** | | | | | **Total** | | | | |
| **Age** | **No of gMG** | **Total persons (mid-year)** | **Prevalence rate** | **95% CI** | | **No of gMG** | **Total persons (mid-year)** | **Prevalence rate** | **95% CI** | | **No of gMG** | **Total persons (mid-year)** | **Prevalence rate** | **95% CI** | |
|  |  |  |  | **LL** | **UL** |  |  |  | **LL** | **UL** |  |  |  | **LL** | **UL** |
| ≤ 9 | 3 | 1,037,873 | 0.29 | 0.06 | 0.84 | 6 | 964,468 | 0.62 | 0.23 | 1.35 | 9 | 2,002,341 | 0.45 | 0.21 | 0.85 |
| 10 – 19 | 14 | 1,207,873 | 1.16 | 0.63 | 1.94 | 28 | 1,105,278 | 2.53 | 1.68 | 3.66 | 42 | 2,313,151 | 1.82 | 1.31 | 2.45 |
| 20 – 29 | 35 | 1,628,763 | 2.15 | 1.50 | 2.99 | 77 | 1,508,267 | 5.11 | 4.03 | 6.38 | 115 | 3,137,030 | 3.67 | 3.03 | 4.40 |
| 30 – 39 | 89 | 1,809,773 | 4.92 | 3.95 | 6.05 | 194 | 1,803,185 | 10.76 | 9.30 | 12.38 | 287 | 3,612,958 | 7.94 | 7.05 | 8.92 |
| 40 – 49 | 179 | 1,833,731 | 9.76 | 8.38 | 11.30 | 281 | 1,895,726 | 14.82 | 13.14 | 16.66 | 461 | 3,729,457 | 12.36 | 11.26 | 13.54 |
| 50 – 59 | 279 | 1,789,353 | 15.59 | 13.82 | 17.53 | 303 | 1,852,755 | 16.35 | 14.56 | 18.30 | 584 | 3,642,108 | 16.03 | 14.76 | 17.39 |
| 60 – 69 | 306 | 1,431,011 | 21.38 | 19.05 | 23.92 | 313 | 1,551,695 | 20.17 | 18.00 | 22.53 | 619 | 2,982,706 | 20.75 | 19.15 | 22.45 |
| 70 – 79 | 169 | 626,183 | 26.99 | 23.07 | 31.38 | 175 | 746,237 | 23.45 | 20.11 | 27.19 | 344 | 1,372,420 | 25.07 | 22.49 | 27.86 |
| ≥ 80 | 84 | 344,490 | 24.38 | 19.45 | 30.19 | 91 | 459,366 | 19.81 | 15.95 | 24.32 | 175 | 803,856 | 21.77 | 18.66 | 25.24 |
| **Total** | 1158 | 1,709,050 | 9.89 | 9.33 | 10.48 | 1470 | 1,886,977 | 12.37 | 11.74 | 13.02 | 2638 | 23,596,027 | 11.18 | 10.76 | 11.61 |

**Table S3** Annual incidence of gMG by calendar year, age and gender in Taiwan per 100,000 population

|  | **2010** | | | | | | | | | | | | | | |
| --- | --- | --- | --- | --- | --- | --- | --- | --- | --- | --- | --- | --- | --- | --- | --- |
|  | **Male** | | | | | **Female** | | | | | **Total** | | | | |
| **Age** | **No of gMG** | **Total persons (mid-year)** | **Incidence rate** | **95% CI** | | **No of gMG** | **Total persons (mid-year)** | **Incidence rate** | **95% CI** | | **No of gMG** | **Total persons (mid-year)** | **Incidence rate** | **95% CI** | |
|  |  |  |  | **LL** | **UL** |  |  |  | **LL** | **UL** |  |  |  | **LL** | **UL** |
| ≤ 19 | 7 | 2,770,993 | 0.25 | 0.10 | 0.52 | 15 | 2,543,412 | 0.59 | 0.33 | 0.97 | 22 | 5,314,405 | 0.41 | 0.26 | 0.63 |
| 20 – 29 | 24 | 1,789,374 | 1.34 | 0.86 | 2.00 | 33 | 1,717,728 | 1.92 | 1.32 | 2.70 | 57 | 3,507,102 | 1.63 | 1.23 | 2.11 |
| 30 – 39 | 33 | 1,895,705 | 1.74 | 1.20 | 2.44 | 61 | 1,908,246 | 3.20 | 2.45 | 4.11 | 95 | 3,803,951 | 2.50 | 2.02 | 3.05 |
| 40 – 49 | 55 | 1,888,270 | 2.91 | 2.19 | 3.79 | 73 | 1,882,181 | 3.88 | 3.04 | 4.88 | 131 | 3,770,451 | 3.47 | 2.90 | 4.12 |
| 50 – 59 | 70 | 1,633,507 | 4.29 | 3.34 | 5.41 | 77 | 1,669,740 | 4.61 | 3.64 | 5.76 | 148 | 3,303,247 | 4.48 | 3.79 | 5.26 |
| 60 – 69 | 60 | 830,321 | 7.23 | 5.51 | 9.30 | 64 | 889,164 | 7.20 | 5.54 | 9.19 | 124 | 1,719,485 | 7.21 | 6.00 | 8.60 |
| 70 – 79 | 53 | 532,608 | 9.95 | 7.45 | 13.02 | 57 | 602,213 | 9.47 | 7.17 | 12.26 | 110 | 1,134,821 | 9.69 | 7.97 | 11.68 |
| ≥ 80 | 20 | 295,202 | 6.78 | 4.14 | 10.46 | 26 | 292,284 | 8.90 | 5.81 | 13.03 | 46 | 587,486 | 7.83 | 5.73 | 10.44 |
| **Total** | 322 | 1,635,980 | 2.77 | 2.47 | 3.09 | 406 | 11,504,968 | 3.53 | 3.19 | 3.89 | 733 | 23,140,948 | 3.17 | 2.94 | 3.41 |

|  | **2011** | | | | | | | | | | | | | | |
| --- | --- | --- | --- | --- | --- | --- | --- | --- | --- | --- | --- | --- | --- | --- | --- |
|  | **Male** | | | | | **Female** | | | | | **Total** | | | | |
| **Age** | **No of gMG** | **Total persons (mid-year)** | **Incidence rate** | **95% CI** | | **No of gMG** | **Total persons (mid-year)** | **Incidence rate** | **95% CI** | | **No of gMG** | **Total persons (mid-year)** | **Prevalence rate** | **95% CI** | |
|  |  |  |  | **LL** | **UL** |  |  |  | **LL** | **UL** |  |  |  | **LL** | **UL** |
| ≤ 19 | 8 | 2,696,496 | 0.30 | 0.13 | 0.58 | 9 | 2,476,750 | 0.36 | 0.17 | 0.69 | 17 | 5,173,246 | 0.33 | 0.19 | 0.53 |
| 20 – 29 | 13 | 1,753,636 | 0.74 | 0.39 | 1.27 | 24 | 1,674,503 | 1.43 | 0.92 | 2.13 | 37 | 3,428,139 | 1.08 | 0.76 | 1.49 |
| 30 – 39 | 31 | 1,911,975 | 1.62 | 1.10 | 2.30 | 63 | 1,932,260 | 3.26 | 2.51 | 4.17 | 96 | 3,844,235 | 2.50 | 2.02 | 3.05 |
| 40 – 49 | 39 | 1,877,616 | 2.08 | 1.48 | 2.84 | 61 | 1,879,939 | 3.24 | 2.48 | 4.17 | 100 | 3,757,555 | 2.66 | 2.17 | 3.24 |
| 50 – 59 | 67 | 1,670,830 | 4.01 | 3.11 | 5.09 | 77 | 1,710,141 | 4.50 | 3.55 | 5.63 | 144 | 3,380,971 | 4.26 | 3.59 | 5.01 |
| 60 – 69 | 64 | 884,889 | 7.23 | 5.57 | 9.24 | 51 | 947,106 | 5.38 | 4.01 | 7.08 | 115 | 1,831,995 | 6.28 | 5.18 | 7.53 |
| 70 – 79 | 35 | 535,632 | 6.53 | 4.55 | 9.09 | 53 | 621,881 | 8.52 | 6.38 | 11.15 | 88 | 1,157,513 | 7.60 | 6.10 | 9.37 |
| ≥ 80 | 26 | 309,376 | 8.40 | 5.49 | 12.31 | 19 | 310,488 | 6.12 | 3.68 | 9.56 | 45 | 619,864 | 7.26 | 5.30 | 9.71 |
| **Total** | 283 | 1,640,450 | 2.43 | 2.16 | 2.73 | 357 | 1,553,068 | 3.09 | 2.78 | 3.43 | 642 | 23,193,518 | 2.77 | 2.56 | 2.99 |

|  | **2012** | | | | | | | | | | | | | | |
| --- | --- | --- | --- | --- | --- | --- | --- | --- | --- | --- | --- | --- | --- | --- | --- |
|  | **Male** | | | | | **Female** | | | | | **Total** | | | | |
| **Age** | **No of gMG** | **Total persons (mid-year)** | **Incidence rate** | **95% CI** | | **No of gMG** | **Total persons (mid-year)** | **Incidence rate** | **95% CI** | | **No of gMG** | **Total persons (mid-year)** | **Incidence rate** | **95% CI** | |
|  |  |  |  | **LL** | **UL** |  |  |  | **LL** | **UL** |  |  |  | **LL** | **UL** |
| ≤ 19 | 10 | 2,641,763 | 0.38 | 0.18 | 0.70 | 7 | 2,428,852 | 0.29 | 0.12 | 0.59 | 17 | 3,011,948 | 0.34 | 0.20 | 0.54 |
| 20 – 29 | 11 | 1,715,440 | 0.64 | 0.32 | 1.15 | 20 | 1,627,987 | 1.23 | 0.75 | 1.90 | 33 | 3,343,427 | 0.99 | 0.68 | 1.39 |
| 30 – 39 | 22 | 1,933,325 | 1.14 | 0.71 | 1.72 | 52 | 1,957,192 | 2.66 | 1.98 | 3.48 | 76 | 3,890,517 | 1.95 | 1.54 | 2.45 |
| 40 – 49 | 43 | 1,859,413 | 2.31 | 1.67 | 3.11 | 60 | 1,870,565 | 3.21 | 2.45 | 4.13 | 103 | 3,729,978 | 2.76 | 2.25 | 3.35 |
| 50 – 59 | 57 | 1,699,874 | 3.35 | 2.54 | 4.34 | 63 | 1,741,936 | 3.62 | 2.78 | 4.63 | 120 | 3,441,810 | 3.49 | 2.89 | 4.17 |
| 60 – 69 | 52 | 949,386 | 5.48 | 4.09 | 7.18 | 39 | 1,016,443 | 3.84 | 2.73 | 5.25 | 92 | 1,965,829 | 4.68 | 3.77 | 5.74 |
| 70 – 79 | 27 | 541,220 | 4.99 | 3.29 | 7.26 | 44 | 639,615 | 6.88 | 5.00 | 9.23 | 71 | 1,180,835 | 6.01 | 4.70 | 7.58 |
| ≥ 80 | 24 | 319,076 | 7.52 | 4.82 | 11.19 | 22 | 328,280 | 6.70 | 4.20 | 10.15 | 46 | 647,356 | 7.11 | 5.20 | 9.48 |
| **Total** | 246 | 1,659,497 | 2.11 | 1.85 | 2.39 | 307 | 1,610,870 | 2.64 | 2.36 | 2.96 | 558 | 23,270,367 | 2.40 | 2.20 | 2.61 |

|  | **2013** | | | | | | | | | | | | | | |
| --- | --- | --- | --- | --- | --- | --- | --- | --- | --- | --- | --- | --- | --- | --- | --- |
|  | **Male** | | | | | **Female** | | | | | **Total** | | | | |
| **Age** | **No of gMG** | **Total persons (mid-year)** | **Incidence rate** | **95% CI** | | **No of gMG** | **Total persons (mid-year)** | **Incidence rate** | **95% CI** | | **No of gMG** | **Total persons (mid-year)** | **Incidence rate** | **95% CI** | |
|  |  |  |  | **LL** | **UL** |  |  |  | **LL** | **UL** |  |  |  | **LL** | **UL** |
| ≤ 19 | 6 | 2,586,403 | 0.23 | 0.09 | 0.50 | 14 | 2,378,974 | 0.59 | 0.32 | 0.99 | 20 | 4,965,377 | 0.40 | 0.25 | 0.62 |
| 20 – 29 | 9 | 1,686,667 | 0.53 | 0.24 | 1.01 | 25 | 1,591,357 | 1.57 | 1.02 | 2.32 | 34 | 3,278,024 | 1.04 | 0.72 | 1.45 |
| 30 – 39 | 36 | 1,950,014 | 1.85 | 1.29 | 2.56 | 58 | 1,975,810 | 2.94 | 2.23 | 3.79 | 96 | 3,925,824 | 2.45 | 1.98 | 2.99 |
| 40 – 49 | 46 | 1,836,680 | 2.50 | 1.83 | 3.34 | 59 | 1,855,392 | 3.18 | 2.42 | 4.10 | 106 | 3,692,072 | 2.87 | 2.35 | 3.47 |
| 50 – 59 | 44 | 1,730,735 | 2.54 | 1.85 | 3.41 | 72 | 1,776,225 | 4.05 | 3.17 | 5.10 | 116 | 3,506,960 | 3.31 | 2.73 | 3.97 |
| 60 – 69 | 43 | 1,013,358 | 4.24 | 3.07 | 5.72 | 61 | 1,085,597 | 5.62 | 4.30 | 7.22 | 104 | 2,098,955 | 4.95 | 4.05 | 6.00 |
| 70 – 79 | 44 | 549,782 | 8.00 | 5.82 | 10.74 | 47 | 656,412 | 7.16 | 5.26 | 9.52 | 91 | 1,206,194 | 7.54 | 6.07 | 9.26 |
| ≥ 80 | 29 | 325,358 | 8.91 | 5.97 | 12.80 | 16 | 345,906 | 4.63 | 2.64 | 7.51 | 45 | 671,264 | 6.70 | 4.89 | 8.97 |
| **Total** | 257 | 1,678,997 | 2.20 | 1.94 | 2.49 | 352 | 11,665,673 | 3.02 | 2.71 | 3.35 | 612 | 23,344,670 | 2.62 | 2.42 | 2.84 |

|  | **2014** | | | | | | | | | | | | | | | | |
| --- | --- | --- | --- | --- | --- | --- | --- | --- | --- | --- | --- | --- | --- | --- | --- | --- | --- |
|  | **Male** | | | | | | **Female** | | | | | | **Total** | | | | |
| **Age** | **No of gMG** | **Total persons (mid-year)** | **Incidence rate** | **95% CI** | | **No of gMG** | | **Total persons (mid-year)** | **Incidence rate** | **95% CI** | | **No of gMG** | | **Total persons (mid-year)** | **Incidence rate** | **95% CI** | |
|  |  |  |  | **LL** | **UL** |  |  |  |  | **LL** | **UL** |  |  |  |  | **LL** | **UL** |
| ≤ 9 | 5 | 1,053,756 | 0.47 | 0.15 | 1.11 | 8 | | 970,675 | 0.82 | 0.36 | 1.62 | 13 | | 2,024,431 | 0.64 | 0.34 | 1.10 |
| 10 – 19 | 4 | 1,471,907 | 0.27 | 0.07 | 0.70 | 12 | | 1,352,990 | 0.89 | 0.46 | 1.55 | 16 | | 2,824,897 | 0.57 | 0.32 | 0.92 |
| 20 – 29 | 16 | 1,665,910 | 0.96 | 0.55 | 1.56 | 34 | | 1,563,666 | 2.17 | 1.51 | 3.04 | 51 | | 3,229,576 | 1.58 | 1.18 | 2.08 |
| 30 – 39 | 30 | 1,959,045 | 1.53 | 1.03 | 2.19 | 55 | | 1,984,951 | 2.77 | 2.09 | 3.61 | 88 | | 3,943,996 | 2.23 | 1.79 | 2.75 |
| 40 – 49 | 43 | 1,814,924 | 2.37 | 1.71 | 3.19 | 54 | | 1,840,949 | 2.93 | 2.20 | 3.83 | 99 | | 3,655,873 | 2.71 | 2.20 | 3.30 |
| 50 – 59 | 70 | 1,757,270 | 3.98 | 3.11 | 5.03 | 58 | | 1,805,384 | 3.21 | 2.44 | 4.15 | 128 | | 3,562,654 | 3.59 | 3.00 | 4.27 |
| 60 – 69 | 83 | 1,080,343 | 7.68 | 6.12 | 9.52 | 50 | | 1,159,340 | 4.31 | 3.20 | 5.69 | 133 | | 2,239,683 | 5.94 | 4.97 | 7.04 |
| 70 – 79 | 36 | 559,088 | 6.44 | 4.51 | 8.91 | 41 | | 670,277 | 6.12 | 4.39 | 8.30 | 77 | | 1,229,365 | 6.26 | 4.94 | 7.83 |
| ≥ 80 | 16 | 329,080 | 4.86 | 2.78 | 7.90 | 25 | | 364,080 | 6.87 | 4.44 | 10.14 | 41 | | 693,160 | 5.91 | 4.24 | 8.02 |
| **Total** | 303 | 1,691,323 | 2.59 | 2.31 | 2.90 | 337 | | 11,712,312 | 2.88 | 2.58 | 3.20 | 646 | | 23,403,635 | 2.76 | 2.55 | 2.98 |

|  | **2015** | | | | | | | | | | | | | | |
| --- | --- | --- | --- | --- | --- | --- | --- | --- | --- | --- | --- | --- | --- | --- | --- |
|  | **Male** | | | | | **Female** | | | | | **Total** | | | | |
| **Age** | **No of gMG** | **Total persons (mid-year)** | **Incidence rate** | **95% CI** | | **No of gMG** | **Total persons (mid-year)** | **Incidence rate** | **95% CI** | | **No of gMG** | **Total persons (mid-year)** | **Incidence rate** | **95% CI** | |
|  |  |  |  | **LL** | **UL** |  |  |  | **LL** | **UL** |  |  |  | **LL** | **UL** |
| ≤ 9 | 3 | 1,053,497 | 0.28 | 0.06 | 0.83 | 3 | 972,459 | 0.31 | 0.06 | 0.90 | 6 | 2,025,956 | 0.30 | 0.11 | 0.64 |
| 10 – 19 | 3 | 1,415,840 | 0.21 | 0.04 | 0.62 | 9 | 1,299,562 | 0.69 | 0.32 | 1.31 | 12 | 2,715,402 | 0.44 | 0.23 | 0.77 |
| 20 – 29 | 17 | 1,654,003 | 1.03 | 0.60 | 1.65 | 24 | 1,544,753 | 1.55 | 1.00 | 2.31 | 41 | 3,198,756 | 1.28 | 0.92 | 1.74 |
| 30 – 39 | 26 | 1,957,524 | 1.33 | 0.87 | 1.95 | 64 | 1,980,834 | 3.23 | 2.49 | 4.13 | 90 | 3,938,358 | 2.29 | 1.84 | 2.81 |
| 40 – 49 | 29 | 1,797,820 | 1.61 | 1.08 | 2.32 | 78 | 1,832,139 | 4.26 | 3.37 | 5.31 | 108 | 3,629,959 | 2.98 | 2.44 | 3.59 |
| 50 – 59 | 77 | 1,773,162 | 4.34 | 3.43 | 5.43 | 66 | 1,824,163 | 3.62 | 2.80 | 4.60 | 143 | 3,597,325 | 3.98 | 3.35 | 4.68 |
| 60 – 69 | 68 | 1,159,208 | 5.87 | 4.56 | 7.44 | 68 | 1,245,763 | 5.46 | 4.24 | 6.92 | 136 | 2,404,971 | 5.65 | 4.74 | 6.69 |
| 70 – 79 | 40 | 561,957 | 7.12 | 5.09 | 9.69 | 46 | 675,113 | 6.81 | 4.99 | 9.09 | 87 | 1,237,070 | 7.03 | 5.63 | 8.67 |
| ≥ 80 | 24 | 331,998 | 7.23 | 4.63 | 10.76 | 19 | 383,119 | 4.96 | 2.99 | 7.74 | 43 | 715,117 | 6.01 | 4.35 | 8.10 |
| **Total** | 287 | 1,705,009 | 2.45 | 2.18 | 2.75 | 377 | 1,757,905 | 3.21 | 2.89 | 3.55 | 666 | 23,462,914 | 2.84 | 2.63 | 3.06 |

|  | **2016** | | | | | | | | | | | | | | |
| --- | --- | --- | --- | --- | --- | --- | --- | --- | --- | --- | --- | --- | --- | --- | --- |
|  | **Male** | | | | | **Female** | | | | | **Total** | | | | |
| **Age** | **No of gMG** | **Total persons (mid-year)** | **Incidence rate** | **95% CI** | | **No of gMG** | **Total persons (mid-year)** | **Incidence rate** | **95% CI** | | **No of gMG** | **Total persons (mid-year)** | **Incidence rate** | **95% CI** | |
|  |  |  |  | **LL** | **UL** |  |  |  | **LL** | **UL** |  |  |  | **LL** | **UL** |
| ≤ 19 | 7 | 2,412,488 | 0.29 | 0.12 | 0.60 | 11 | 2,219,648 | 0.50 | 0.25 | 0.89 | 18 | 4,632,136 | 0.39 | 0.23 | 0.61 |
| 20 – 29 | 9 | 1,657,980 | 0.54 | 0.25 | 1.03 | 16 | 1,542,308 | 1.04 | 0.59 | 1.68 | 25 | 3,200,288 | 0.78 | 0.51 | 1.15 |
| 30 – 39 | 29 | 1,924,375 | 1.51 | 1.01 | 2.16 | 58 | 1,944,493 | 2.98 | 2.26 | 3.86 | 88 | 3,868,868 | 2.27 | 1.82 | 2.80 |
| 40 – 49 | 34 | 1,797,783 | 1.89 | 1.31 | 2.64 | 66 | 1,839,170 | 3.59 | 2.78 | 4.57 | 100 | 3,636,953 | 2.75 | 2.24 | 3.34 |
| 50 – 59 | 74 | 1,781,772 | 4.15 | 3.26 | 5.21 | 72 | 1,836,712 | 3.92 | 3.07 | 4.94 | 147 | 3,618,484 | 4.06 | 3.43 | 4.77 |
| 60 – 69 | 73 | 1,244,742 | 5.86 | 4.60 | 7.37 | 66 | 1,339,750 | 4.93 | 3.81 | 6.27 | 139 | 2,584,492 | 5.38 | 4.52 | 6.35 |
| 70 – 79 | 32 | 562,531 | 5.69 | 3.89 | 8.03 | 40 | 676,889 | 5.91 | 4.22 | 8.05 | 72 | 1,239,420 | 5.81 | 4.55 | 7.32 |
| ≥ 80 | 16 | 333,988 | 4.79 | 2.74 | 7.78 | 22 | 401,316 | 5.48 | 3.44 | 8.30 | 38 | 735,304 | 5.17 | 3.66 | 7.09 |
| **Total** | 274 | 11,715,659 | 2.34 | 2.07 | 2.63 | 351 | 11,800,286 | 2.97 | 2.67 | 3.30 | 627 | 23,515,945 | 2.67 | 2.46 | 2.88 |

|  | **2017** | | | | | | | | | | | | | | |
| --- | --- | --- | --- | --- | --- | --- | --- | --- | --- | --- | --- | --- | --- | --- | --- |
|  | **Male** | | | | | **Female** | | | | | **Total** | | | | |
| **Age** | **No of gMG** | **Total persons (mid-year)** | **Incidence rate** | **95% CI** | | **No of gMG** | **Total persons (mid-year)** | **Incidence rate** | **95% CI** | | **No of gMG** | **Total persons (mid-year)** | **Incidence rate** | **95% CI** | |
|  |  |  |  | **LL** | **UL** |  |  |  | **LL** | **UL** |  |  |  | **LL** | **UL** |
| ≤ 29 | 20 | 4,018,618 | 0.50 | 0.30 | 0.77 | 38 | 3,711,653 | 1.02 | 0.72 | 1.41 | 58 | 7,730,271 | 0.75 | 0.57 | 0.97 |
| 30 – 39 | 23 | 1,879,059 | 1.22 | 0.78 | 1.84 | 36 | 1,892,798 | 1.90 | 1.33 | 2.63 | 62 | 3,771,857 | 1.64 | 1.26 | 2.11 |
| 40 – 49 | 39 | 1,808,930 | 2.16 | 1.53 | 2.95 | 71 | 1,858,061 | 3.82 | 2.98 | 4.82 | 111 | 3,666,991 | 3.03 | 2.49 | 3.65 |
| 50 – 59 | 65 | 1,786,986 | 3.64 | 2.81 | 4.64 | 51 | 1,844,418 | 2.77 | 2.06 | 3.64 | 116 | 3,631,404 | 3.19 | 2.64 | 3.83 |
| 60 – 69 | 85 | 1,316,924 | 6.45 | 5.16 | 7.98 | 75 | 1,421,204 | 5.28 | 4.15 | 6.61 | 160 | 2,738,128 | 5.84 | 4.97 | 6.82 |
| 70 – 79 | 50 | 572,847 | 8.73 | 6.48 | 11.51 | 39 | 688,478 | 5.66 | 4.03 | 7.74 | 89 | 1,261,325 | 7.06 | 5.67 | 8.68 |
| ≥ 80 | 23 | 336,061 | 6.84 | 4.34 | 10.27 | 27 | 419,485 | 6.44 | 4.24 | 9.36 | 50 | 755,546 | 6.62 | 4.91 | 8.72 |
| **Total** | 305 | 11,719,425 | 2.60 | 2.32 | 2.91 | 337 | 1,836,097 | 2.85 | 2.55 | 3.17 | 646 | 23,555,522 | 2.74 | 2.54 | 2.96 |

|  | **2018** | | | | | | | | | | | | | | |
| --- | --- | --- | --- | --- | --- | --- | --- | --- | --- | --- | --- | --- | --- | --- | --- |
|  | **Male** | | | | | **Female** | | | | | **Total** | | | | |
| **Age** | **No of gMG** | **Total persons (mid-year)** | **Incidence rate** | **95% CI** | | **No of gMG** | **Total persons (mid-year)** | **Incidence rate** | **95% CI** | | **No of gMG** | **Total persons (mid-year)** | **Incidence rate** | **95% CI** | |
|  |  |  |  | **LL** | **UL** |  |  |  | **LL** | **UL** |  |  |  | **LL** | **UL** |
| ≤ 19 | 9 | 2,294,754 | 0.39 | 0.18 | 0.74 | 19 | 2,113,465 | 0.90 | 0.54 | 1.40 | 28 | 4,408,219 | 0.64 | 0.42 | 0.92 |
| 20 – 29 | 16 | 1,653,946 | 0.97 | 0.55 | 1.57 | 29 | 1,533,181 | 1.89 | 1.27 | 2.72 | 46 | 3,187,127 | 1.44 | 1.06 | 1.93 |
| 30 – 39 | 35 | 1,848,096 | 1.89 | 1.32 | 2.63 | 39 | 1,851,811 | 2.11 | 1.50 | 2.88 | 79 | 3,699,907 | 2.14 | 1.69 | 2.66 |
| 40 – 49 | 40 | 1,818,995 | 2.20 | 1.57 | 2.99 | 55 | 1,875,770 | 2.93 | 2.21 | 3.82 | 96 | 3,694,765 | 2.60 | 2.10 | 3.17 |
| 50 – 59 | 63 | 1,789,423 | 3.52 | 2.71 | 4.50 | 63 | 1,849,071 | 3.41 | 2.62 | 4.36 | 126 | 3,638,494 | 3.46 | 2.88 | 4.12 |
| 60 – 69 | 110 | 1,376,599 | 7.99 | 6.57 | 9.63 | 72 | 1,489,570 | 4.83 | 3.78 | 6.09 | 183 | 2,866,169 | 6.38 | 5.49 | 7.38 |
| 70 – 79 | 42 | 594,860 | 7.06 | 5.09 | 9.54 | 39 | 712,093 | 5.48 | 3.89 | 7.49 | 81 | 1,306,953 | 6.20 | 4.92 | 7.70 |
| ≥ 80 | 17 | 339,574 | 5.01 | 2.92 | 8.02 | 24 | 438,872 | 5.47 | 3.50 | 8.14 | 41 | 778,446 | 5.27 | 3.78 | 7.15 |
| **Total** | 332 | 11,716,247 | 2.83 | 2.54 | 3.16 | 340 | 11,863,833 | 2.87 | 2.57 | 3.19 | 680 | 23,580,080 | 2.88 | 2.67 | 3.11 |

|  | **2019** | | | | | | | | | | | | | | |
| --- | --- | --- | --- | --- | --- | --- | --- | --- | --- | --- | --- | --- | --- | --- | --- |
|  | **Male** | | | | | **Female** | | | | | **Total** | | | | |
| **Age** | **No of gMG** | **Total persons (mid-year)** | **Incidence rate** | **95% CI** | | **No of gMG** | **Total persons (mid-year)** | **Incidence rate** | **95% CI** | | **No of gMG** | **Total persons (mid-year)** | **Incidence rate** | **95% CI** | |
|  |  |  |  | **LL** | **UL** |  |  |  | **LL** | **UL** |  |  |  | **LL** | **UL** |
| ≤ 19 | 5 | 2,245,746 | 0.22 | 0.07 | 0.52 | 9 | 2,069,746 | 0.43 | 0.20 | 0.83 | 14 | 4,315,492 | 0.32 | 0.18 | 0.54 |
| 20 – 29 | 15 | 1,628,763 | 0.92 | 0.52 | 1.52 | 26 | 1,508,267 | 1.72 | 1.13 | 2.53 | 43 | 3,137,030 | 1.37 | 0.99 | 1.85 |
| 30 – 39 | 26 | 1,809,773 | 1.44 | 0.94 | 2.11 | 53 | 1,803,185 | 2.94 | 2.20 | 3.84 | 79 | 3,612,958 | 2.19 | 1.73 | 2.73 |
| 40 – 49 | 53 | 1,833,731 | 2.89 | 2.17 | 3.78 | 61 | 1,895,726 | 3.22 | 2.46 | 4.13 | 114 | 3,729,457 | 3.06 | 2.52 | 3.67 |
| 50 – 59 | 76 | 1,789,353 | 4.25 | 3.35 | 5.32 | 63 | 1,852,755 | 3.40 | 2.61 | 4.35 | 140 | 3,642,108 | 3.84 | 3.23 | 4.54 |
| 60 – 69 | 87 | 1,431,011 | 6.08 | 4.87 | 7.50 | 85 | 1,551,695 | 5.48 | 4.38 | 6.77 | 172 | 2,982,706 | 5.77 | 4.94 | 6.70 |
| 70 – 79 | 47 | 626,183 | 7.51 | 5.52 | 9.98 | 44 | 746,237 | 5.90 | 4.28 | 7.92 | 91 | 1,372,420 | 6.63 | 5.34 | 8.14 |
| ≥ 80 | 24 | 344,490 | 6.97 | 4.46 | 10.37 | 17 | 459,366 | 3.70 | 2.16 | 5.93 | 41 | 803,856 | 5.10 | 3.66 | 6.92 |
| **Total** | 333 | 1,709,050 | 2.84 | 2.55 | 3.17 | 360 | 11,886,977 | 3.03 | 2.72 | 3.36 | 696 | 23,596,027 | 2.95 | 2.73 | 3.18 |

**Table S4** Annual all-cause fatality during the study period (2009 – 2019) per 100 patients

|  | **2009** | | | | | | | | | | | | | | | |
| --- | --- | --- | --- | --- | --- | --- | --- | --- | --- | --- | --- | --- | --- | --- | --- | --- |
|  | **Male** | | | | | **Female** | | | | | | **Total** | | | | |
| **Age** | **No of death** | **Total gMG** | **Fatality rate** | **95% CI** | | **No of death** | **Total gMG** | **Fatality rate** | **95% CI** | | **No of death** | | **Total gMG** | **Fatality rate** | **95% CI** | |
|  |  |  |  | **LL** | **UL** |  |  |  | **LL** | **UL** |  |  |  |  | **LL** | **UL** |
| ≤ 9 | 0 | 12 | 0.00 | 0.00 | 26.46 | 0 | 8 | 0.00 | 0.00 | 36.94 | 0 | | 20 | 0.00 | 0.00 | 16.84 |
| 10 – 19 | 0 | 9 | 0.00 | 0.00 | 33.63 | 0 | 19 | 0.00 | 0.00 | 17.65 | 0 | | 28 | 0.00 | 0.00 | 12.34 |
| 20 – 29 | 0 | 35 | 0.00 | 0.00 | 10.00 | 0 | 79 | 0.00 | 0.00 | 4.56 | 0 | | 117 | 0.00 | 0.00 | 3.10 |
| 30 – 39 | 2 | 78 | 2.56 | 0.31 | 8.96 | 2 | 139 | 1.44 | 0.17 | 5.10 | 4 | | 220 | 1.82 | 0.50 | 4.59 |
| 40 – 49 | 1 | 146 | 0.68 | 0.02 | 3.76 | 5 | 193 | 2.59 | 0.85 | 5.94 | 6 | | 339 | 1.77 | 0.65 | 3.81 |
| 50 – 59 | 6 | 147 | 4.08 | 1.51 | 8.67 | 3 | 180 | 1.67 | 0.35 | 4.79 | 9 | | 328 | 2.74 | 1.26 | 5.14 |
| 60 – 69 | 9 | 113 | 7.96 | 3.71 | 14.58 | 5 | 134 | 3.73 | 1.22 | 8.49 | 14 | | 247 | 5.67 | 3.13 | 9.33 |
| 70 – 79 | 9 | 107 | 8.41 | 3.92 | 15.37 | 4 | 94 | 4.26 | 1.17 | 10.54 | 13 | | 201 | 6.47 | 3.49 | 10.81 |
| ≥ 80 | 2 | 35 | 5.71 | 0.70 | 19.16 | 7 | 41 | 17.07 | 7.15 | 32.06 | 9 | | 76 | 11.84 | 5.56 | 21.29 |
| **Total** | 29 | 682 | 4.25 | 2.87 | 6.05 | 26 | 887 | 2.93 | 1.92 | 4.27 | 55 | | 1576 | 3.49 | 2.64 | 4.52 |

|  | **2010** | | | | | | | | | | | | | | | | |
| --- | --- | --- | --- | --- | --- | --- | --- | --- | --- | --- | --- | --- | --- | --- | --- | --- | --- |
|  | **Male** | | | | | | **Female** | | | | | | **Total** | | | | |
| **Age** | **No of death** | **Total gMG** | **Fatality rate** | **95% CI** | | **No of death** | | **Total gMG** | **Fatality rate** | **95% CI** | | **No of death** | | **Total gMG** | **Fatality rate** | **95% CI** | |
|  |  |  |  | **LL** | **UL** |  |  |  |  | **LL** | **UL** |  |  |  |  | **LL** | **UL** |
| ≤ 9 | 0 | 7 | 0.00 | 0.00 | 40.96 | 0 | | 12 | 0.00 | 0.00 | 26.46 | 0 | | 19 | 0.00 | 0.00 | 17.65 |
| 10 – 19 | 0 | 9 | 0.00 | 0.00 | 33.63 | 0 | | 14 | 0.00 | 0.00 | 23.16 | 0 | | 23 | 0.00 | 0.00 | 14.82 |
| 20 – 29 | 0 | 40 | 0.00 | 0.00 | 8.81 | 0 | | 76 | 0.00 | 0.00 | 4.74 | 0 | | 118 | 0.00 | 0.00 | 3.08 |
| 30 – 39 | 0 | 78 | 0.00 | 0.00 | 4.62 | 0 | | 158 | 0.00 | 0.00 | 2.31 | 0 | | 237 | 0.00 | 0.00 | 1.54 |
| 40 – 49 | 2 | 136 | 1.47 | 0.18 | 5.21 | 3 | | 197 | 1.52 | 0.32 | 4.39 | 5 | | 336 | 1.49 | 0.48 | 3.44 |
| 50 – 59 | 3 | 167 | 1.80 | 0.37 | 5.16 | 4 | | 203 | 1.97 | 0.54 | 4.97 | 7 | | 372 | 1.88 | 0.76 | 3.84 |
| 60 – 69 | 4 | 128 | 3.13 | 0.86 | 7.81 | 6 | | 146 | 4.11 | 1.52 | 8.73 | 10 | | 274 | 3.65 | 1.76 | 6.61 |
| 70 – 79 | 5 | 117 | 4.27 | 1.40 | 9.69 | 5 | | 108 | 4.63 | 1.52 | 10.47 | 10 | | 225 | 4.44 | 2.15 | 8.02 |
| ≥ 80 | 6 | 41 | 14.63 | 5.57 | 29.17 | 9 | | 52 | 17.31 | 8.23 | 30.33 | 15 | | 93 | 16.13 | 9.32 | 25.20 |
| **Total** | 20 | 723 | 2.77 | 1.70 | 4.24 | 27 | | 966 | 2.80 | 1.85 | 4.04 | 47 | | 1697 | 2.77 | 2.04 | 3.67 |

|  | **2011** | | | | | | | | | | | | | | | | |
| --- | --- | --- | --- | --- | --- | --- | --- | --- | --- | --- | --- | --- | --- | --- | --- | --- | --- |
|  | **Male** | | | | | | **Female** | | | | | | **Total** | | | | |
| **Age** | **No of death** | **Total gMG** | **Fatality rate** | **95% CI** | | **No of death** | | **Total gMG** | **Fatality rate** | **95% CI** | | **No of death** | | **Total gMG** | **Fatality rate** | **95% CI** | |
|  |  |  |  | **LL** | **UL** |  |  |  |  | **LL** | **UL** |  |  |  |  | **LL** | **UL** |
| ≤ 9 | 0 | 5 | 0.00 | 0.00 | 52.18 | 0 | | 9 | 0.00 | 0.00 | 33.63 | 0 | | 14 | 0.00 | 0.00 | 23.16 |
| 10 – 19 | 0 | 11 | 0.00 | 0.00 | 28.49 | 0 | | 17 | 0.00 | 0.00 | 19.51 | 0 | | 28 | 0.00 | 0.00 | 12.34 |
| 20 – 29 | 0 | 36 | 0.00 | 0.00 | 9.74 | 0 | | 70 | 0.00 | 0.00 | 5.13 | 0 | | 107 | 0.00 | 0.00 | 3.39 |
| 30 – 39 | 1 | 82 | 1.22 | 0.03 | 6.61 | 0 | | 177 | 0.00 | 0.00 | 2.06 | 1 | | 262 | 0.38 | 0.01 | 2.11 |
| 40 – 49 | 4 | 140 | 2.86 | 0.78 | 7.15 | 3 | | 195 | 1.54 | 0.32 | 4.43 | 7 | | 335 | 2.09 | 0.84 | 4.26 |
| 50 – 59 | 10 | 191 | 5.24 | 2.54 | 9.42 | 3 | | 211 | 1.42 | 0.29 | 4.10 | 13 | | 402 | 3.23 | 1.73 | 5.47 |
| 60 – 69 | 7 | 143 | 4.90 | 1.99 | 9.83 | 6 | | 143 | 4.20 | 1.56 | 8.91 | 13 | | 287 | 4.53 | 2.43 | 7.62 |
| 70 – 79 | 9 | 113 | 7.96 | 3.71 | 14.58 | 11 | | 137 | 8.03 | 4.08 | 13.91 | 20 | | 250 | 8.00 | 4.95 | 12.09 |
| ≥ 80 | 7 | 55 | 12.73 | 5.27 | 24.48 | 7 | | 53 | 13.21 | 5.48 | 25.34 | 14 | | 108 | 12.96 | 7.27 | 20.79 |
| **Total** | 38 | 776 | 4.90 | 3.49 | 6.66 | 30 | | 1012 | 2.96 | 2.01 | 4.21 | 68 | | 1793 | 3.79 | 2.96 | 4.78 |

|  | **2012** | | | | | | | | | | | | | | | | |
| --- | --- | --- | --- | --- | --- | --- | --- | --- | --- | --- | --- | --- | --- | --- | --- | --- | --- |
|  | **Male** | | | | | | **Female** | | | | | | **Total** | | | | |
| **Age** | **No of death** | **Total gMG** | **Fatality rate** | **95% CI** | | **No of death** | | **Total gMG** | **Fatality rate** | **95% CI** | | **No of death** | | **Total gMG** | **Fatality rate** | **95% CI** | |
|  |  |  |  | **LL** | **UL** |  |  |  |  | **LL** | **UL** |  |  |  |  | **LL** | **UL** |
| ≤ 9 | 0 | 7 | 0.00 | 0.00 | 40.96 | 1 | | 6 | 16.67 | 0.42 | 64.12 | 1 | | 13 | 7.69 | 0.19 | 36.03 |
| 10 – 19 | 0 | 11 | 0.00 | 0.00 | 28.49 | 0 | | 13 | 0.00 | 0.00 | 24.71 | 0 | | 24 | 0.00 | 0.00 | 14.25 |
| 20 – 29 | 1 | 35 | 2.86 | 0.07 | 14.92 | 0 | | 62 | 0.00 | 0.00 | 5.78 | 1 | | 100 | 1.00 | 0.03 | 5.45 |
| 30 – 39 | 1 | 76 | 1.32 | 0.03 | 7.11 | 0 | | 183 | 0.00 | 0.00 | 2.00 | 1 | | 262 | 0.38 | 0.01 | 2.11 |
| 40 – 49 | 2 | 135 | 1.48 | 0.18 | 5.25 | 0 | | 192 | 0.00 | 0.00 | 1.90 | 2 | | 328 | 0.61 | 0.07 | 2.19 |
| 50 – 59 | 4 | 192 | 2.08 | 0.57 | 5.25 | 5 | | 219 | 2.28 | 0.75 | 5.25 | 9 | | 411 | 2.19 | 1.01 | 4.12 |
| 60 – 69 | 3 | 138 | 2.17 | 0.45 | 6.22 | 5 | | 135 | 3.70 | 1.21 | 8.43 | 8 | | 275 | 2.91 | 1.26 | 5.65 |
| 70 – 79 | 4 | 114 | 3.51 | 0.96 | 8.74 | 7 | | 126 | 5.56 | 2.26 | 11.11 | 11 | | 240 | 4.58 | 2.31 | 8.05 |
| ≥ 80 | 5 | 63 | 7.94 | 2.63 | 17.56 | 11 | | 58 | 18.97 | 9.87 | 31.41 | 16 | | 121 | 13.22 | 7.75 | 20.58 |
| **Total** | 20 | 771 | 2.59 | 1.59 | 3.98 | 29 | | 994 | 2.92 | 1.96 | 4.16 | 49 | | 1774 | 2.76 | 2.05 | 3.64 |

|  | **2013** | | | | | | | | | | | | | | | | |
| --- | --- | --- | --- | --- | --- | --- | --- | --- | --- | --- | --- | --- | --- | --- | --- | --- | --- |
|  | **Male** | | | | | | **Female** | | | | | | **Total** | | | | |
| **Age** | **No of death** | **Total gMG** | **Fatality rate** | **95% CI** | | **No of death** | | **Total gMG** | **Fatality rate** | **95% CI** | | **No of death** | | **Total gMG** | **Fatality rate** | **95% CI** | |
|  |  |  |  | **LL** | **UL** |  |  |  |  | **LL** | **UL** |  |  |  |  | **LL** | **UL** |
| ≤ 9 | 0 | 5 | 0.00 | 0.00 | 52.18 | 0 | | 5 | 0.00 | 0.00 | 52.18 | 0 | | 10 | 0.00 | 0.00 | 30.85 |
| 10 – 19 | 0 | 10 | 0.00 | 0.00 | 30.85 | 0 | | 21 | 0.00 | 0.00 | 16.11 | 0 | | 31 | 0.00 | 0.00 | 11.22 |
| 20 – 29 | 0 | 28 | 0.00 | 0.00 | 12.34 | 0 | | 61 | 0.00 | 0.00 | 5.87 | 0 | | 89 | 0.00 | 0.00 | 4.06 |
| 30 – 39 | 0 | 96 | 0.00 | 0.00 | 3.77 | 0 | | 190 | 0.00 | 0.00 | 1.92 | 0 | | 291 | 0.00 | 0.00 | 1.26 |
| 40 – 49 | 1 | 146 | 0.68 | 0.02 | 3.76 | 4 | | 206 | 1.94 | 0.53 | 4.90 | 5 | | 354 | 1.41 | 0.46 | 3.27 |
| 50 – 59 | 6 | 188 | 3.19 | 1.18 | 6.82 | 5 | | 237 | 2.11 | 0.69 | 4.85 | 11 | | 425 | 2.59 | 1.30 | 4.58 |
| 60 – 69 | 5 | 148 | 3.38 | 1.11 | 7.71 | 3 | | 178 | 1.69 | 0.35 | 4.85 | 8 | | 328 | 2.44 | 1.06 | 4.75 |
| 70 – 79 | 7 | 131 | 5.34 | 2.18 | 10.70 | 3 | | 142 | 2.11 | 0.44 | 6.05 | 10 | | 273 | 3.66 | 1.77 | 6.63 |
| ≥ 80 | 16 | 72 | 22.22 | 13.27 | 33.56 | 8 | | 51 | 15.69 | 7.02 | 28.59 | 24 | | 123 | 19.51 | 12.92 | 27.63 |
| **Total** | 35 | 824 | 4.25 | 2.98 | 5.86 | 23 | | 1091 | 2.11 | 1.34 | 3.15 | 58 | | 1924 | 3.01 | 2.30 | 3.88 |

|  | **2014** | | | | | | | | | | | | | | |
| --- | --- | --- | --- | --- | --- | --- | --- | --- | --- | --- | --- | --- | --- | --- | --- |
|  | **Male** | | | | | **Female** | | | | | **Total** | | | | |
| **Age** | **No of death** | **Total gMG** | **Fatality rate** | **95% CI** | | **No of death** | **Total gMG** | **Fatality rate** | **95% CI** | | **No of death** | **Total gMG** | **Fatality rate** | **95% CI** | |
|  |  |  |  | **LL** | **UL** |  |  |  | **LL** | **UL** |  |  |  | **LL** | **UL** |
| ≤ 9 | 0 | 6 | 0.00 | 0.00 | 45.93 | 0 | 9 | 0.00 | 0.00 | 33.63 | 0 | 15 | 0.00 | 0.00 | 21.80 |
| 10 – 19 | 0 | 10 | 0.00 | 0.00 | 30.85 | 0 | 24 | 0.00 | 0.00! | 14.25 | 0 | 34 | 0.00 | 0.00 | 10.28 |
| 20 – 29 | 0 | 36 | 0.00 | 0.00 | 9.74 | 0 | 80 | 0.00 | 0.00 | 4.51 | 0 | 117 | 0.00 | 0.00 | 3.10 |
| 30 – 39 | 2 | 101 | 1.98 | 0.24 | 6.97 | 2 | 210 | 0.95 | 0.12 | 3.40 | 4 | 316 | 1.27 | 0.35 | 3.21 |
| 40 – 49 | 3 | 144 | 2.08 | 0.43 | 5.97 | 1 | 209 | 0.48 | 0.01 | 2.64 | 4 | 357 | 1.12 | 0.31 | 2.84 |
| 50 – 59 | 7 | 228 | 3.07 | 1.24 | 6.22 | 2 | 240 | 0.83 | 0.10 | 2.98 | 9 | 468 | 1.92 | 0.88 | 3.62 |
| 60 – 69 | 8 | 205 | 3.90 | 1.70 | 7.54 | 8 | 190 | 4.21 | 1.84 | 8.13 | 16 | 396 | 4.04 | 2.33 | 6.48 |
| 70 – 79 | 10 | 125 | 8.00 | 3.90 | 14.22 | 9 | 136 | 6.62 | 3.07 | 12.19 | 19 | 261 | 7.28 | 4.44 | 11.13 |
| ≥ 80 | 11 | 68 | 16.18 | 8.36 | 27.10 | 8 | 69 | 11.59 | 5.14 | 21.57 | 19 | 137 | 13.87 | 8.56 | 20.81 |
| **Total** | 41 | 923 | 4.44 | 3.21 | 5.98 | 30 | 1167 | 2.57 | 1.74 | 3.65 | 71 | 2101 | 3.38 | 2.65 | 4.24 |

|  | **2015** | | | | | | | | | | | | | | | | |
| --- | --- | --- | --- | --- | --- | --- | --- | --- | --- | --- | --- | --- | --- | --- | --- | --- | --- |
|  | **Male** | | | | | | **Female** | | | | | | **Total** | | | | |
| **Age** | **No of death** | **Total gMG** | **Fatality rate** | **95% CI** | | **No of death** | | **Total gMG** | **Fatality rate** | **95% CI** | | **No of death** | | **Total gMG** | **Fatality rate** | **95% CI** | |
|  |  |  |  | **LL** | **UL** |  |  |  |  | **LL** | **UL** |  |  |  |  | **LL** | **UL** |
| ≤ 9 | 0 | 9 | 0.00 | 0.00 | 33.63 | 0 | | 6 | 0.00 | 0.00 | 45.93 | 0 | | 15 | 0.00 | 0.00 | 21.80 |
| 10 – 19 | 0 | 10 | 0.00 | 0.00 | 30.85 | 0 | | 20 | 0.00 | 0.00 | 16.84 | 0 | | 30 | 0.00 | 0.00 | 11.57 |
| 20 – 29 | 0 | 34 | 0.00 | 0.00 | 10.28 | 0 | | 59 | 0.00 | 0.00 | 6.06 | 0 | | 93 | 0.00 | 0.00 | 3.89 |
| 30 – 39 | 2 | 94 | 2.13 | 0.26 | 7.48 | 0 | | 209 | 0.00 | 0.00 | 1.75 | 2 | | 305 | 0.66 | 0.08 | 2.35 |
| 40 – 49 | 1 | 137 | 0.73 | 0.02 | 4.00 | 3 | | 234 | 1.28 | 0.27 | 3.70 | 4 | | 373 | 1.07 | 0.29 | 2.72 |
| 50 – 59 | 5 | 229 | 2.18 | 0.71 | 5.02 | 7 | | 259 | 2.70 | 1.09 | 5.49 | 12 | | 488 | 2.46 | 1.28 | 4.26 |
| 60 – 69 | 7 | 231 | 3.03 | 1.23 | 6.14 | 7 | | 218 | 3.21 | 1.30 | 6.50 | 14 | | 450 | 3.11 | 1.71 | 5.16 |
| 70 – 79 | 5 | 113 | 4.42 | 1.45 | 10.02 | 6 | | 145 | 4.14 | 1.53 | 8.79 | 11 | | 259 | 4.25 | 2.14 | 7.47 |
| ≥ 80 | 8 | 64 | 12.50 | 5.55 | 23.15 | 18 | | 75 | 24.00 | 14.89 | 35.25 | 26 | | 139 | 18.71 | 12.60 | 26.19 |
| **Total** | 28 | 921 | 3.04 | 2.03 | 4.36 | 41 | | 1225 | 3.35 | 2.41 | 4.51 | 69 | | 2152 | 3.21 | 2.50 | 4.04 |

|  | **2016** | | | | | | | | | | | | | | | | |
| --- | --- | --- | --- | --- | --- | --- | --- | --- | --- | --- | --- | --- | --- | --- | --- | --- | --- |
|  | **Male** | | | | | | **Female** | | | | | | **Total** | | | | |
| **Age** | **No of death** | **Total gMG** | **Fatality rate** | **95% CI** | | **No of death** | | **Total gMG** | **Fatality rate** | **95% CI** | | **No of death** | | **Total gMG** | **Fatality rate** | **95% CI** | |
|  |  |  |  | **LL** | **UL** |  |  |  |  | **LL** | **UL** |  |  |  |  | **LL** | **UL** |
| ≤ 9 | 0 | 9 | 0.00 | 0.00 | 33.63 | 0 | | 5 | 0.00 | 0.00 | 52.18 | 0 | | 14 | 0.00 | 0.00 | 23.16 |
| 10 – 19 | 0 | 10 | 0.00 | 0.00 | 30.85 | 0 | | 25 | 0.00 | 0.00 | 13.72 | 0 | | 35 | 0.00 | 0.00 | 10.00 |
| 20 – 29 | 0 | 32 | 0.00 | 0.00 | 10.89 | 0 | | 59 | 0.00 | 0.00 | 6.06 | 0 | | 91 | 0.00 | 0.00 | 3.97 |
| 30 – 39 | 0 | 99 | 0.00 | 0.00 | 3.66 | 2 | | 213 | 0.94 | 0.11 | 3.35 | 2 | | 314 | 0.64 | 0.08 | 2.28 |
| 40 – 49 | 2 | 136 | 1.47 | 0.18 | 5.21 | 4 | | 240 | 1.67 | 0.46 | 4.21 | 6 | | 378 | 1.59 | 0.58 | 3.42 |
| 50 – 59 | 7 | 243 | 2.88 | 1.17 | 5.84 | 5 | | 282 | 1.77 | 0.58 | 4.09 | 12 | | 527 | 2.28 | 1.18 | 3.94 |
| 60 – 69 | 8 | 238 | 3.36 | 1.46 | 6.52 | 4 | | 231 | 1.73 | 0.47 | 4.37 | 12 | | 471 | 2.55 | 1.32 | 4.41 |
| 70 – 79 | 6 | 126 | 4.76 | 1.77 | 10.08 | 5 | | 146 | 3.42 | 1.12 | 7.81 | 11 | | 273 | 4.03 | 2.03 | 7.10 |
| ≥ 80 | 9 | 56 | 16.07 | 7.62 | 28.33 | 20 | | 88 | 22.73 | 14.47 | 32.89 | 29 | | 144 | 20.14 | 13.92 | 27.63 |
| **Total** | 32 | 949 | 3.37 | 2.32 | 4.73 | 40 | | 1289 | 3.10 | 2.23 | 4.20 | 72 | | 2247 | 3.20 | 2.52 | 4.02 |

|  | **2017** | | | | | | | | | | | | | | | | |
| --- | --- | --- | --- | --- | --- | --- | --- | --- | --- | --- | --- | --- | --- | --- | --- | --- | --- |
|  | **Male** | | | | | | **Female** | | | | | | **Total** | | | | |
| **Age** | **No of death** | **Total gMG** | **Fatality rate** | **95% CI** | | **No of death** | | **Total gMG** | **Fatality rate** | **95% CI** | | **No of death** | | **Total gMG** | **Fatality rate** | **95% CI** | |
|  |  |  |  | **LL** | **UL** |  |  |  |  | **LL** | **UL** |  |  |  |  | **LL** | **UL** |
| ≤ 9 | 0 | 4 | 0.00 | 0.00 | 60.24 | 0 | | 6 | 0.00 | 0.00 | 45.93 | 0 | | 10 | 0.00 | 0.00 | 30.85 |
| 10 – 19 | 0 | 9 | 0.00 | 0.00 | 33.63 | 0 | | 26 | 0.00 | 0.00 | 13.23 | 0 | | 35 | 0.00 | 0.00 | 10.00 |
| 20 – 29 | 2 | 38 | 5.26 | 0.64 | 17.75 | 0 | | 71 | 0.00 | 0.00 | 5.06 | 2 | | 109 | 1.83 | 0.22 | 6.47 |
| 30 – 39 | 1 | 97 | 1.03 | 0.03 | 5.61 | 1 | | 181 | 0.55 | 0.01 | 3.04 | 2 | | 281 | 0.71 | 0.09 | 2.55 |
| 40 – 49 | 2 | 144 | 1.39 | 0.17 | 4.93 | 0 | | 259 | 0.00 | 0.00 | 1.41 | 2 | | 405 | 0.49 | 0.06 | 1.77 |
| 50 – 59 | 6 | 244 | 2.46 | 0.91 | 5.28 | 5 | | 288 | 1.74 | 0.57 | 4.00 | 11 | | 532 | 2.07 | 1.04 | 3.67 |
| 60 – 69 | 7 | 270 | 2.59 | 1.05 | 5.27 | 6 | | 256 | 2.34 | 0.86 | 5.03 | 13 | | 526 | 2.47 | 1.32 | 4.19 |
| 70 – 79 | 10 | 146 | 6.85 | 3.33 | 12.24 | 8 | | 147 | 5.44 | 2.38 | 10.44 | 18 | | 293 | 6.14 | 3.68 | 9.54 |
| ≥ 80 | 4 | 71 | 5.63 | 1.56 | 13.80 | 21 | | 88 | 23.86 | 15.42 | 34.14 | 25 | | 159 | 15.72 | 10.44 | 22.33 |
| **Total** | 32 | 1023 | 3.13 | 2.15 | 4.39 | 41 | | 1322 | 3.10 | 2.23 | 4.18 | 73 | | 2350 | 3.11 | 2.44 | 3.89 |

|  | **2018** | | | | | | | | | | | | | | | | |
| --- | --- | --- | --- | --- | --- | --- | --- | --- | --- | --- | --- | --- | --- | --- | --- | --- | --- |
|  | **Male** | | | | | | **Female** | | | | | | **Total** | | | | |
| **Age** | **No of death** | **Total gMG** | **Fatality rate** | **95% CI** | | **No of death** | | **Total gMG** | **Fatality rate** | **95% CI** | | **No of death** | | **Total gMG** | **Fatality rate** | **95% CI** | |
|  |  |  |  | **LL** | **UL** |  |  |  |  | **LL** | **UL** |  |  |  |  | **LL** | **UL** |
| ≤ 9 | 0 | 4 | 0.00 | 0.00 | 60.24 | 0 | | 8 | 0.00 | 0.00 | 36.94 | 0 | | 12 | 0.00 | 0.00 | 26.46 |
| 10 – 19 | 0 | 17 | 0.00 | 0.00 | 19.51 | 0 | | 31 | 0.00 | 0.00 | 11.22 | 0 | | 48 | 0.00 | 0.00 | 7.40 |
| 20 – 29 | 0 | 38 | 0.00 | 0.00 | 9.25 | 1 | | 77 | 1.30 | 0.03 | 7.02 | 1 | | 116 | 0.86 | 0.02 | 4.71 |
| 30 – 39 | 1 | 101 | 0.99 | 0.03 | 5.39 | 2 | | 186 | 1.08 | 0.13 | 3.83 | 3 | | 293 | 1.02 | 0.21 | 2.96 |
| 40 – 49 | 2 | 167 | 1.20 | 0.15 | 4.26 | 3 | | 259 | 1.16 | 0.24 | 3.35 | 5 | | 428 | 1.17 | 0.38 | 2.71 |
| 50 – 59 | 5 | 243 | 2.06 | 0.67 | 4.74 | 3 | | 297 | 1.01 | 0.21 | 2.92 | 8 | | 540 | 1.48 | 0.64 | 2.90 |
| 60 – 69 | 13 | 308 | 4.22 | 2.27 | 7.11 | 8 | | 277 | 2.89 | 1.25 | 5.61 | 21 | | 587 | 3.58 | 2.23 | 5.42 |
| 70 – 79 | 17 | 153 | 11.11 | 6.61 | 17.19 | 14 | | 170 | 8.24 | 4.58 | 13.43 | 31 | | 323 | 9.60 | 6.61 | 13.35 |
| ≥ 80 | 13 | 78 | 16.67 | 9.18 | 26.81 | 10 | | 95 | 10.53 | 5.16 | 18.51 | 23 | | 174 | 13.22 | 8.57 | 19.17 |
| **Total** | 51 | 1109 | 4.60 | 3.44 | 6.00 | 41 | | 1400 | 2.93 | 2.11 | 3.95 | 92 | | 2521 | 3.65 | 2.95 | 4.46 |

|  | **2019** | | | | | | | | | | | | | | | |
| --- | --- | --- | --- | --- | --- | --- | --- | --- | --- | --- | --- | --- | --- | --- | --- | --- |
|  | **Male** | | | | | **Female** | | | | | | **Total** | | | | |
| **Age** | **No of death** | **Total gMG** | **Fatality rate** | **95% CI** | | **No of death** | **Total gMG** | **Fatality rate** | **95% CI** | | **No of death** | | **Total gMG** | **Fatality rate** | **95% CI** | |
|  |  |  |  | **LL** | **UL** |  |  |  | **LL** | **UL** |  |  |  |  | **LL** | **UL** |
| ≤ 9 | 0 | 3 | 0.00 | 0.00 | 70.76 | 0 | 6 | 0.00 | 0.00 | 45.93 | 0 | | 9 | 0.00 | 0.00 | 33.63 |
| 10 – 19 | 0 | 14 | 0.00 | 0.00 | 23.16 | 0 | 28 | 0.00 | 0.00 | 12.34 | 0 | | 42 | 0.00 | 0.00 | 8.41 |
| 20 – 29 | 0 | 35 | 0.00 | 0.00 | 10.00 | 0 | 77 | 0.00 | 0.00 | 4.68 | 0 | | 115 | 0.00 | 0.00! | 3.16 |
| 30 – 39 | 2 | 89 | 2.25 | 0.27 | 7.88 | 1 | 194 | 0.52 | 0.01 | 2.84 | 3 | | 287 | 1.05 | 0.22 | 3.02 |
| 40 – 49 | 1 | 179 | 0.56 | 0.01 | 3.07 | 1 | 281 | 0.36 | 0.01 | 1.97 | 2 | | 461 | 0.43 | 0.05 | 1.56 |
| 50 – 59 | 4 | 279 | 1.43 | 0.39 | 3.63 | 4 | 303 | 1.32 | 0.36 | 3.35 | 8 | | 584 | 1.37 | 0.59 | 2.68 |
| 60 – 69 | 6 | 306 | 1.96 | 0.72 | 4.22 | 13 | 313 | 4.15 | 2.23 | 7.00 | 19 | | 619 | 3.07 | 1.86 | 4.75 |
| 70 – 79 | 12 | 169 | 7.10 | 3.72 | 12.07 | 10 | 175 | 5.71 | 2.77 | 10.26 | 22 | | 344 | 6.40 | 4.05 | 9.52 |
| ≥ 80 | 13 | 84 | 15.48 | 8.51 | 25.01 | 11 | 91 | 12.09 | 6.19 | 20.60 | 24 | | 175 | 13.71 | 8.99 | 19.72 |
| **Total** | 38 | 1158 | 3.28 | 2.33 | 4.48 | 40 | 1470 | 2.72 | 1.95 | 3.69 | 78 | | 2638 | 2.96 | 2.34 | 3.68 |
